# A cardiovascular, craniofacial, and neurodevelopmental disorder caused by loss-of-function variants in the eIF3 complex component genes *EIF3A* and *EIF3B*

**DOI:** 10.1016/j.ajhg.2026.02.005

**Published:** 2026-02-11

**Authors:** Esra Erkut, Cherith Somerville, Marci L.B. Schwartz, Laura McDonald, Qiliang Ding, Olivia M. Moran, Xin Chen, Roozbeh Manshaei, Anne-Sophie Riedijk, Marie-Therese Schnürer, Daniel C. Koboldt, Stylianos E. Antonarakis, Emma C. Bedoukian, Xavier Blanc, Laura K. Conlin, Helen Cox, Karin E.M. Diderich, Bri Dingmann, Christèle Dubourg, Frances Elmslie, Luis F. Escobar, Rachel Gosselin, Maria J. Guillen Sacoto, Cynthia D. Haag, Lisa Herzig, Ramanand Jeeneea, Priti Kenia, Konstantinos Kolokotronis, Anna M. Kopps, Christin Kupper, Hayley Lees, Jacqueline Leonard, Jonathan Levy, Rebecca Littlejohn, Demian Mayer, Kim L. McBride, Scott D. McLean, Nikhil Pattani, Laurence Perrin, Véronique Pingault, Chloé Quelin, Emmanuelle Ranza, Anita Rauch, Sara L. Reichert, Joana Rosmaninho-Salgado, Cara Skraban, Sérgio Sousa, Melissa Stuebben, Paolo Zanoni, Raymond H. Kim, Ian C. Scott, Rebekah K. Jobling

## Main text

(The American Journal of Human Genetics *112*, 2625–2642; November 6, 2025)

In the originally published version of this article, Dr. Kim L. McBride, who submitted phenotype information for Proband 6 and was on the original ACMG platform presentation, was accidentally not included on the manuscript. This author has now been included in the author list.

The authors regret this error.

