# Supplementary material for: A cardiovascular, craniofacial, and neurodevelopmental disorder caused by loss-of-function variants in the eIF3 complex component genes EIF3A and EIF3B
Source: Am J Hum Genet. 2025 Sep 30;112(11):2625–42. doi: 10.1016/j.ajhg.2025.09.008 (PMC12808965; doi:10.1016/j.ajhg.2025.09.008)
Supplement: Document S1. Figures S1–S7 and Tables S3 and S4 [file mmc1.pdf]

The American Journal of Human Genetics, Volume *112*

Supplemental information

A cardiovascular, craniofacial, and neurodevelopmental disorder caused by loss-of-function variants in the eIF3 complex component genes *EIF3A* and *EIF3B*

Esra Erkut, Cherith Somerville, Marci L.B. Schwartz, Laura McDonald, Qiliang Ding, Olivia M. Moran, Xin Chen, Roozbeh Manshaei, Anne-Sophie Riedijk, Marie-Therese Schnürer, Daniel C. Koboldt, Stylianos E. Antonarakis, Emma C. Bedoukian, Xavier Blanc, Laura K. Conlin, Helen Cox, Karin E.M. Diderich, Bri Dingmann, Christèle Dubourg, Frances Elmslie, Luis F. Escobar, Rachel Gosselin, Maria J. Guillen Sacoto, Cynthia D. Haag, Lisa Herzig, Ramanand Jeeneea, Priti Kenia, Konstantinos Kolokotronis, Anna M. Kopps, Christin Kupper, Hayley Lees, Jacqueline Leonard, Jonathan Levy, Rebecca Littlejohn, Demian Mayer, Scott D. McLean, Nikhil Pattani, Laurence Perrin, Véronique Pingault, Chloé Quelin, Emmanuelle Ranza, Anita Rauch, Sara L. Reichert, Joana Rosmaninho-Salgado, Cara Skraban, Sérgio Sousa, Melissa Stuebben, Paolo Zanoni, Raymond H. Kim, Ian C. Scott, and Rebekah K. Jobling

Supplemental Information

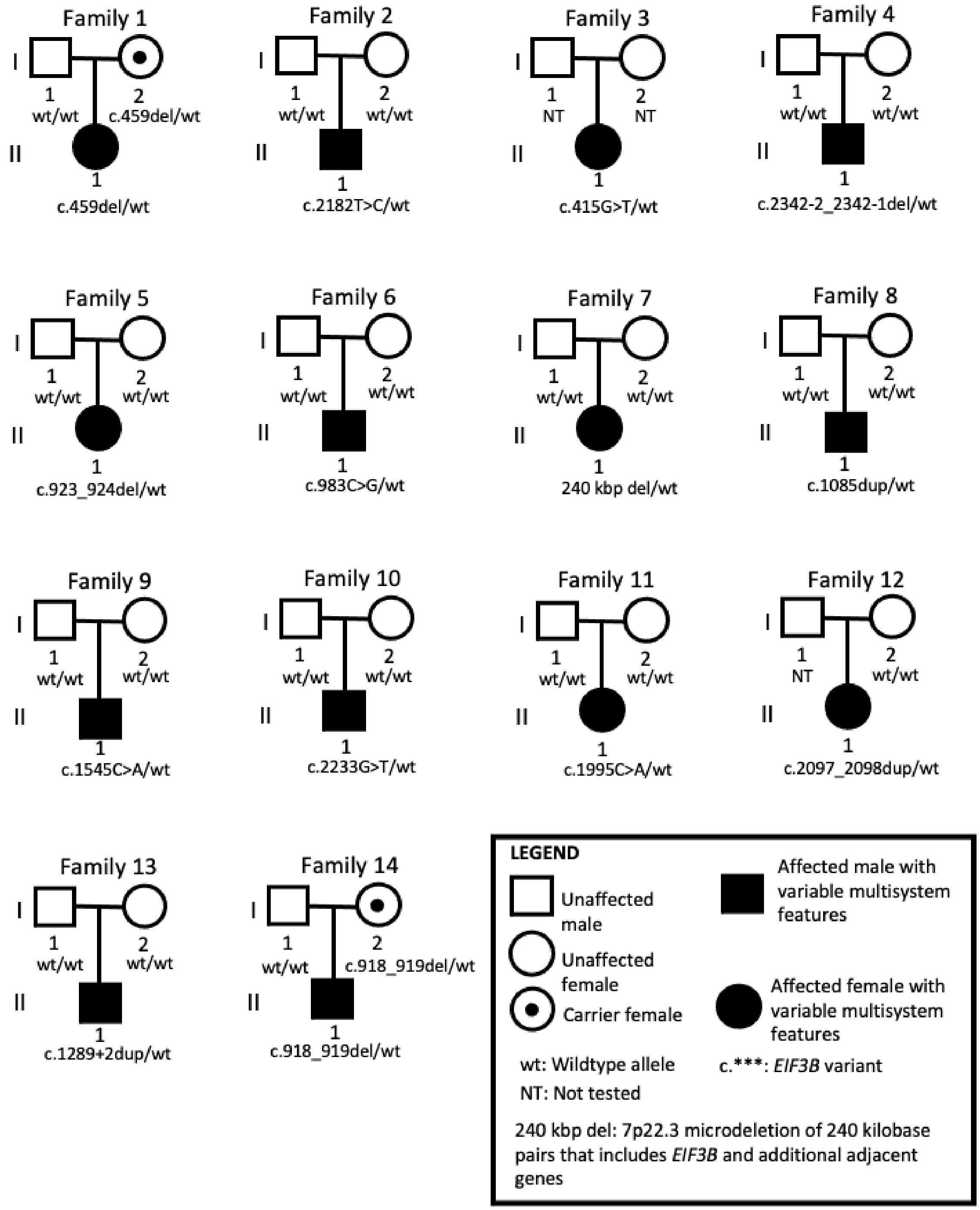

Figure S1. Family pedigrees for individuals with *EIF3B* variants. Solid black symbols represent affected family members with variable multisystem features, primarily including craniofacial differences, heart anomalies, and mild neurodevelopmental symptoms. A dot in the center indicates variant carriers who do not present with clinical symptoms. Family numbers correspond to the proband numbers in the main text and in Tables 2-3.

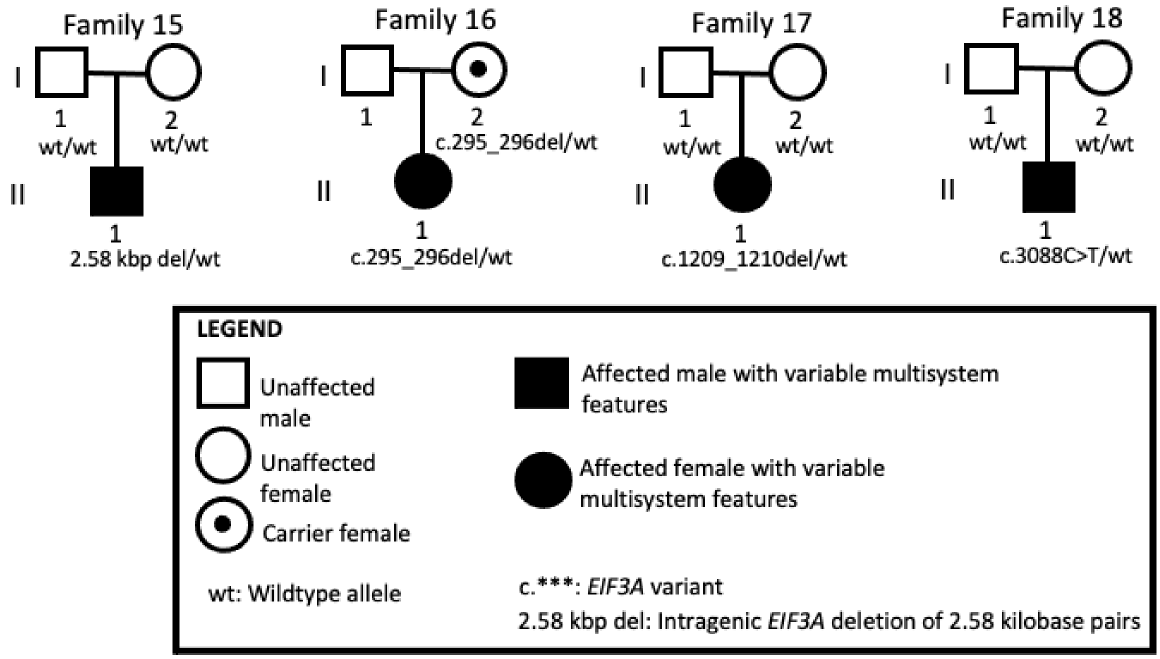

Figure S2. Family pedigrees for individuals with *EIF3A* variants. Solid black symbols represent affected family members with variable multisystem features, primarily including craniofacial differences, heart anomalies, and mild neurodevelopmental symptoms. A dot in the center indicates variant carriers who do not present with clinical symptoms. Family numbers correspond to the proband numbers in the main text and in Tables 2-3.

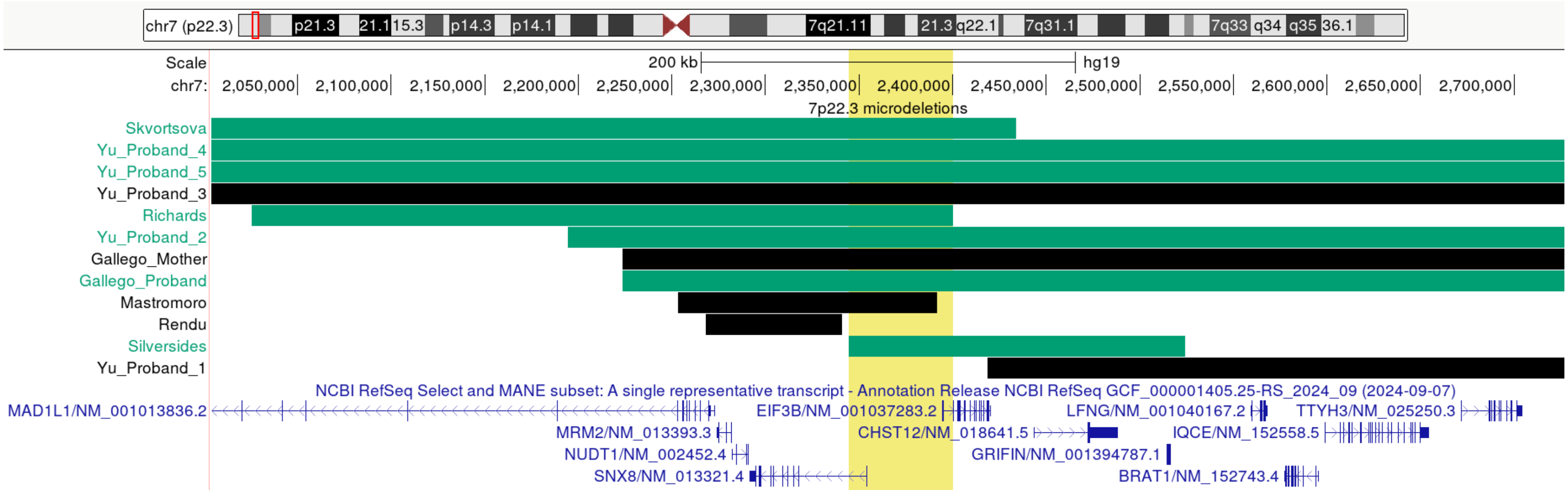

Figure S3. Overlap of 7p22.3 microdeletions reported in the literature. Previously reported deletions overlapping the 7p22.3 region[1-7] are summarized in Table S2. Deletions observed in individuals with cardiac abnormalities (n=7) are marked in green, while those in individuals without cardiac abnormalities are in black. Yellow vertical lines indicate the smallest overlapping region associated with the cardiac phenotype, which includes *EIF3B.* Mapping and gene content were generated using UCSC Genome Custom Tracks Tool[8].

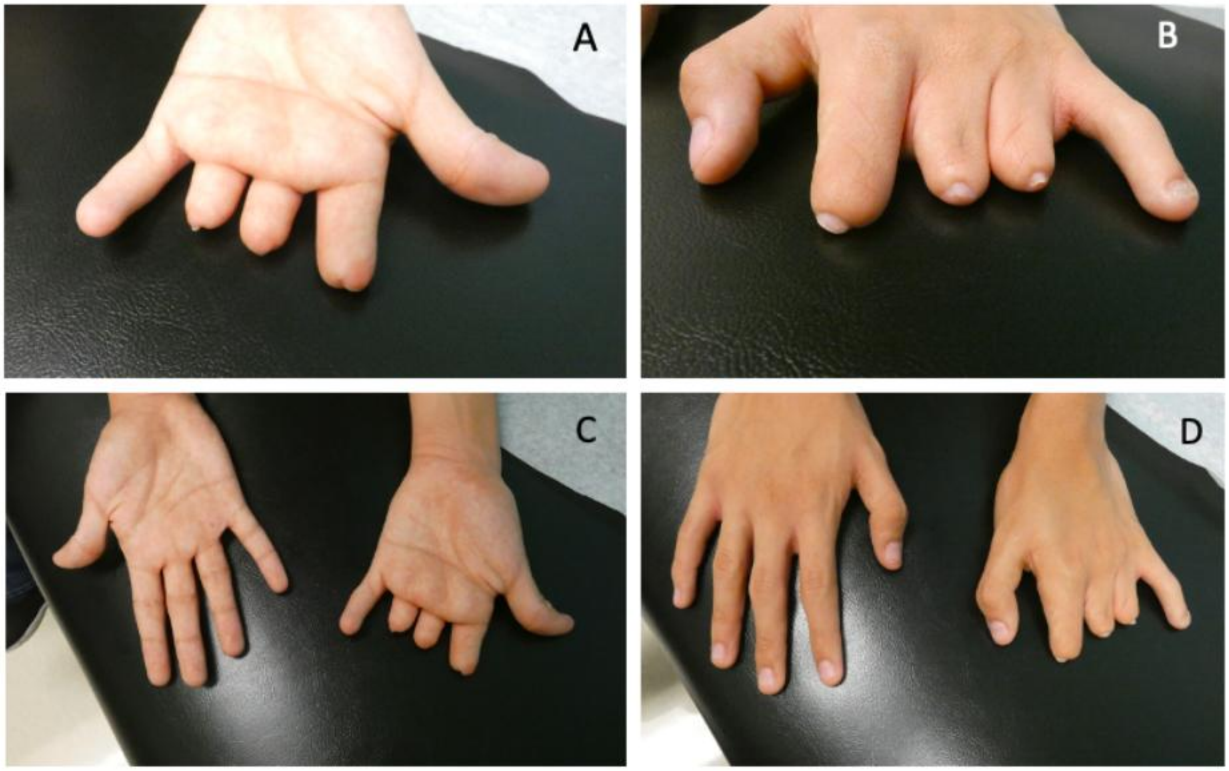

Figure S4 (A-D) Images of the hands of proband #9 presenting with transverse terminal deficiency of the left hand affecting digits 2 through 4 at the distal aspects of the proximal phalanges, with preservation of rudimentary nails.

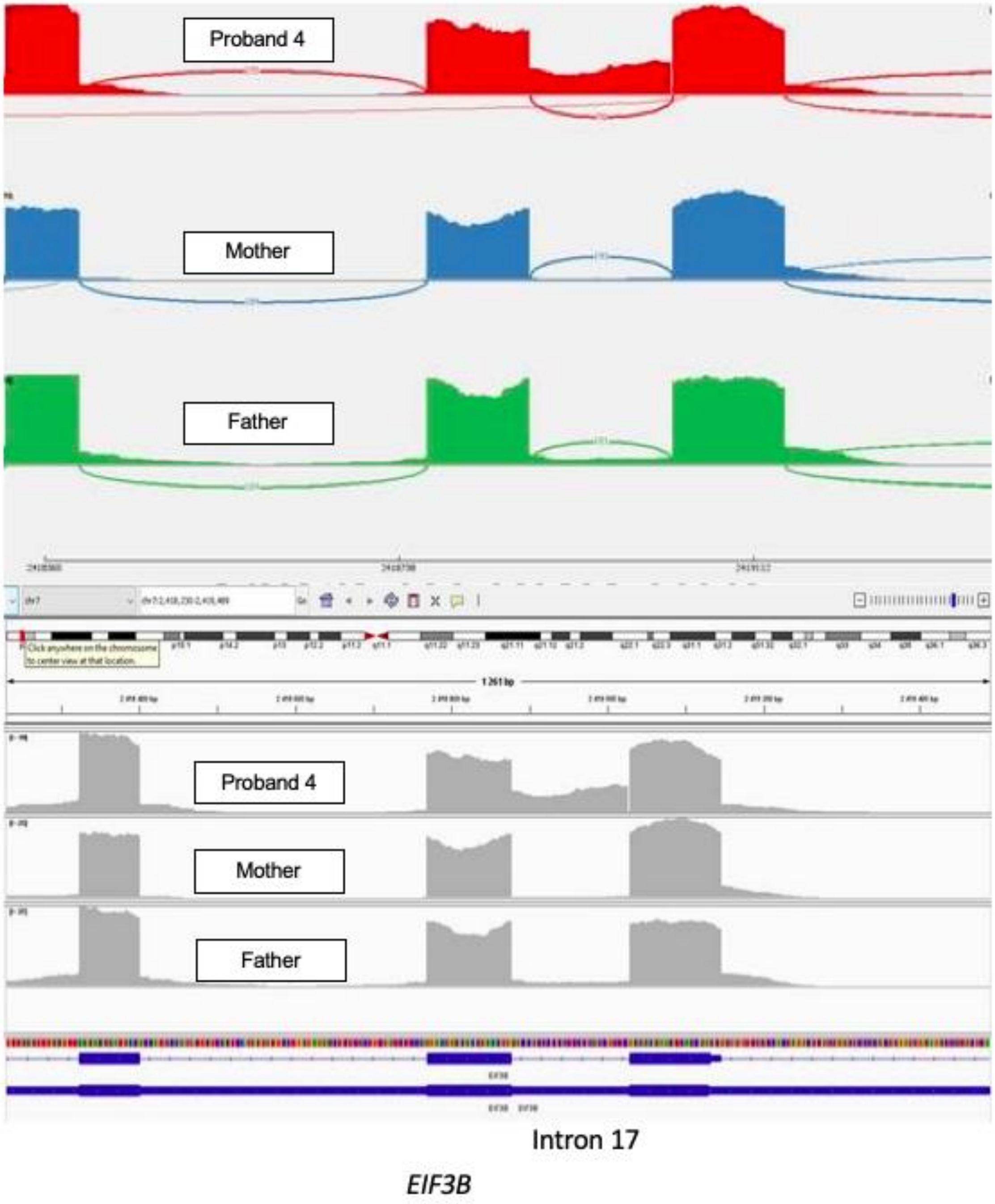

Figure S5: Sashimi plot and coverage graph from IGV for RNA-sequencing results of c.2342-2_2342-1del in proband #4, as well as their mother and father. RNA-seq was performed following identification of a de novo variant via next-generation sequencing (c.2342-2_2342-1del). The sequencing results show intron 17 retention in the proband 4, leading to a readthrough intron starting at amino acid 782 and causing a frameshift. The 2bp deletion is also visible, confirming that intron retention comes from the mutated allele only.

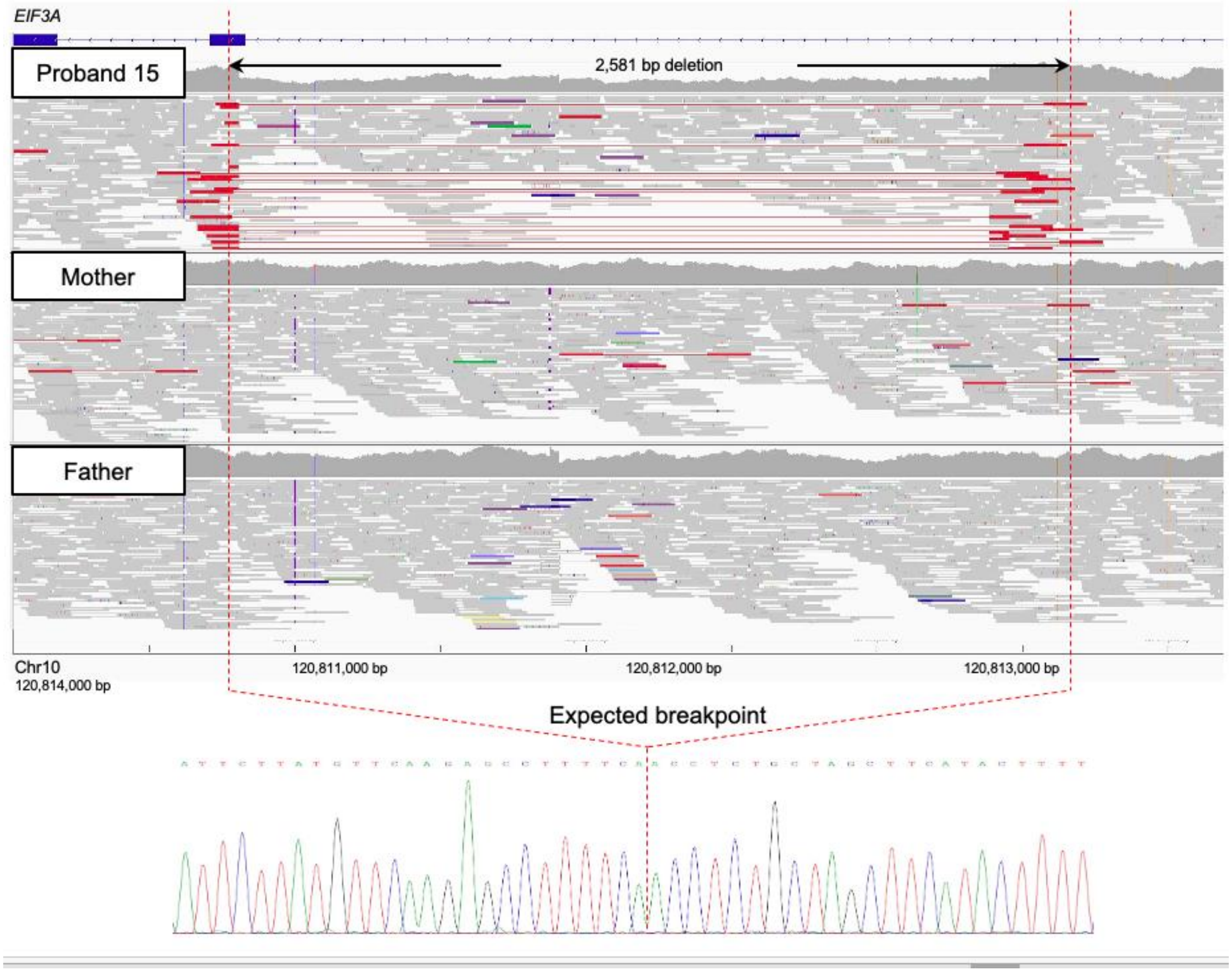

Figure S6: Sanger sequencing validation of the 2.58 kilobase pairs intragenic *EIF3A* deletion in proband #15. IGV browser view of the BAM file for proband #15 and the proband's mother and father, showing the Sanger track confirming the breakpoint. The genomic coordinates for this intragenic deletion are chr10:119051299-119053879 (GRCh38); (chr10:120810811-120813391 [GRCh37]). Sanger validation confirmed that the variant is de novo, with the 5' breakpoint located in intron 14 and the 3' breakpoint extending 23 base pairs into the 5'end of exon 15, disrupting the canonical acceptor splice site of exon 15.

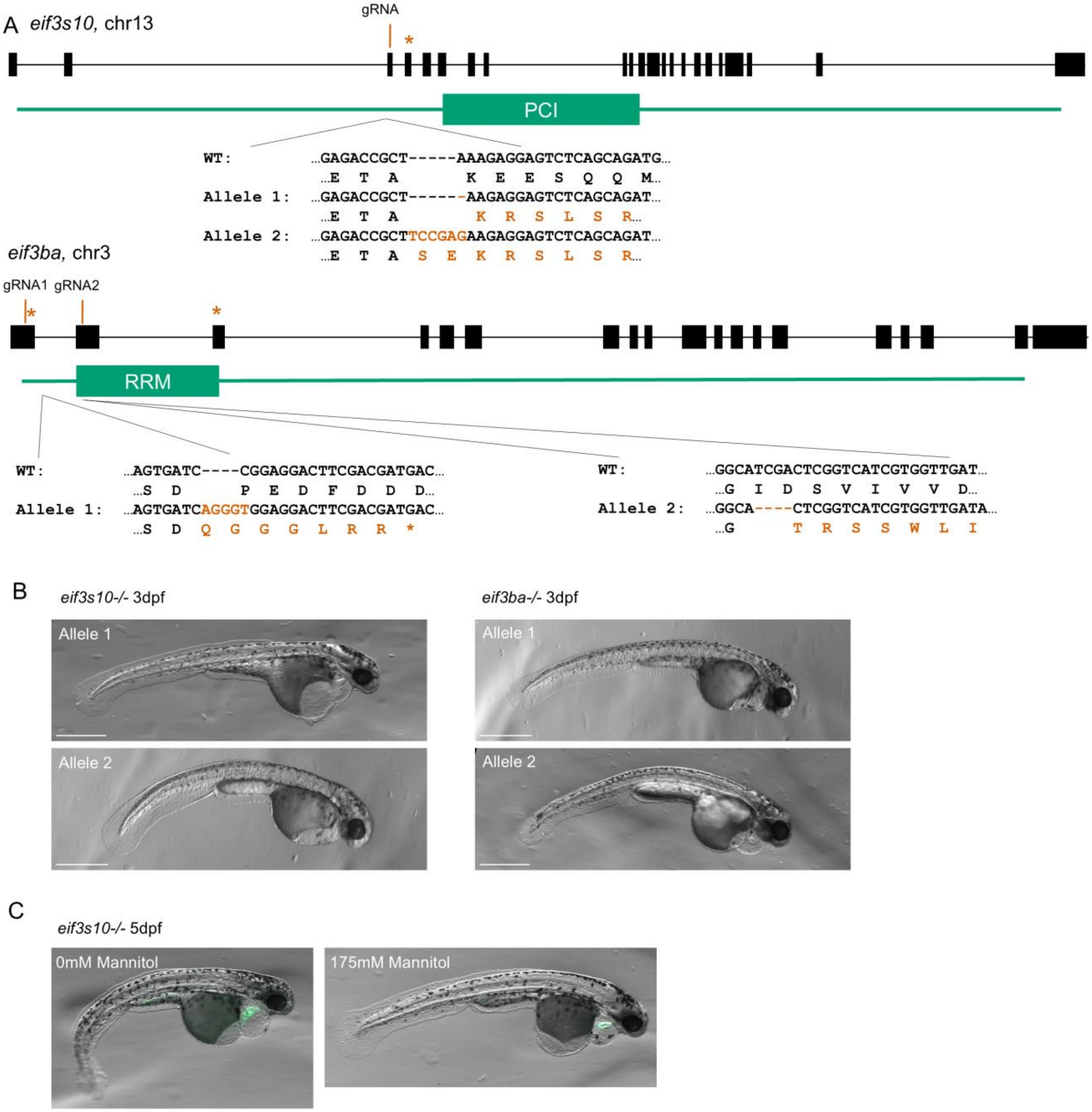

Figure S7: Zebrafish mutant models for *eif3s10* and *eif3ba* exhibit cardiac defects (A) schematic of *eif3s10* and *eif3ba* null mutant generation using the CRISPR/Cas9 system. Black boxes indicate exons, drawn to scale. Orange letters indicate indels generated, and asterisks represent a premature stop codon, which occurs before or inside the critical protein domains (schematized in green). (B) Two different CRISPR alleles for *eif3s10* and *eif3ba* demonstrate the same phenotype. (C) Raising homozygous mutant embryos in 175mM mannitol salts to balance osmolarity did not alleviate the stretched heart phenotype, indicating a primary defect in heart development.

Proband	Gene	GenBank Transcripts	Variant	Zygosity; inheritance	Disease Association	Inheritance Pattern	Classification; criteria applied[a]	Additional evidence
2	*KCNK4* (MIM: 605720)	NM_033310.3; NP_201567.1	c.724G>A, p.(Gly242Ser)	Heterozygous	Facial dysmorphism, hypertrichosis, epilepsy, intellectual disability/developmental delay, and gingival overgrowth syndrome (MIM: 618381)	AD	VUS: PM6, PP3	Identified as *de novo* in this individual; Present in 7 alleles in gnomAD V4.1.0; Not reported in the literature.
2	*PLOD1* (MIM: 153454)	NM_000302.4; NP_000293.2	c.244A>T, p.(Lys82*)	Heterozygous	Kyphoscoliotic type 1 Ehlers-Danlos syndrome (MIM: 225400)	AR	Likely pathogenic: PVS1, PM2	Present in 1 allele in gnomaD V4.1.0; Not reported in the literature. Biallelic loss-of-function in the *PLOD1* gene is an established mechanism of disease.
5	*GJB2* (MIM: 121011)	NM_004004.6; NP_003995.2	c.35delG, p.(Gly12Valfs*2)	Heterozygous	Deafness (MIM: 601544, 220290)	AR, AD	Pathogenic for AR hearing loss: PVS1, PM3_VS, PS4, BA1	ClinVar has an entry for this variant (VCV000017004); classified as pathogenic by the ClinGen Hearing Loss Variant Curation Expert Panel.
5	*TMPRSS3* (MIM: 605511)	NM_001256317.3; NP_001243246.1	c.413C>A, p.(Ala138Glu)	Heterozygous	Deafness (MIM: 601072)	AR	Pathogenic: PS4, PM3_VS, PP1_Strong	ClinVar has an entry for this variant (VCV000046119).
6	*BRAF* (MIM: 164757)	NM_001374258.1; NP_001361187.1	c.1325C>G, p.(Pro442Arg)	Heterozygous; maternal	Noonan syndrome 7 (MIM: 613706)	AD	VUS: No criteria applied (per BRAF RASopathy	Identified in 15 alleles in gnomAD V4.1.0. Not found in a known critical

							ClinGen guidelines)	domain. ClinVar has an entry for this variant (VCV000560678). Not reported in the literature.
6	*ZMIZ1* (MIM: 607159)	NM_020338.4; NP_065071.1	c.767G>C, p.(Gly256Ala)	Heterozygous; maternal	Neurodevelopmental disorder with dysmorphic facies and distal skeletal anomalies (MIM: 618659)	AD	VUS: No criteria applied.	Identified in 9 alleles tested from gnomAD V4.1.0. ClinVar has an entry for this variant (VCV002640635). Not reported in the literature.
6	*MED13* (MIM: 618009)	NM_005121.3; NP_005112.2	c.3676C>T, p.(Arg1226Cys)	Heterozygous; paternal	Intellectual developmental disorder (MIM: 618009)	AD	VUS: BP1	Identified in 28 alleles tested from gnomAD V4.1.0. ClinVar has an entry for this variant (VCV003293987). Not reported in the literature.
18	*SHOC2* (MIM: 602775)	NM_007373.3; NP_031399.2	c.126A>T, p.(Glu42Asp)	Heterozygous; maternal. Also inherited in twin brother.	Noonan syndrome-like with loose anagen hair 1 (MIM: 607721)	AD	VUS: PM2_Supporting, BP4	Absent in gnomAD v4.1.0. ClinVar has an entry for this variant (VCV002200060). Not reported in the literature

Table S3: Additional genetic findings in individuals in the cohort. [a] Classification and criteria based on Richards et al.[9]. Abbreviations: AD: autosomal dominant. AR: Autosomal recessive.

Gene	pLI	LEOUF
EIF3B (MIM: 603917)	1	0.11
AMZ1 (MIM: 615168)	0	1.88
AP5Z1 (MIM: 613653)	0	1.64
BRAT1 (MIM: 614506)	0	1.39
CARD11 (MIM: 607210)	1	0.36
CHST12 (MIM: 610129)	0	1.12
FOXK1 (MIM: 616302)	0.05	0.66
GNA12 (MIM: 604394)	0.93	0.56
GRIFIN (MIM: 619187)	0	1.89
IQCE (MIM: 617631)	0	1.44
LFNG (MIM: 602576)	0.2	0.76
*MAD1L1 (*MIM: 602686)	0	1.23
MMD2 (MIM: 624581)	0	1.35
MRM2 (MIM: 606906)	0	1.72
NUDT1 (MIM: 600312)	0	1.74
PAPOLB (MIM: 607436)	0	1.05
RADIL (MIM: 611491)	0	1.49
RBAK (MIM: 608191)	0	0.75
SDK1 (MIM: 607216)	0	0.77
SNX8 (MIM: 614905)	0	0.74
TTYH3 (MIM: 608919)	0.01	0.68

Table S4. Loss-of-function constraint metrics for genes in the 7p22.3 region. The Probability of being loss-of-function intolerant (pLI) and loss-of-function observed/expected upper bound fraction scores (LEOUF) were obtained from gnomAD v4.1.0. Genes in the 7p22.3 region considered constrained against loss-of-function variation include *EIF3B* and *CARD11*.

References (Table S1-S4)

1. Gallego, C.J., Grant, J., Mikhail, F.M., Barger, C., Robin, N.H. (2010). Use of array comparative genome hybridization in orofacial clefting. J Craniofac Surg. *21*(5), 1591–1594. doi: 10.1097/SCS.0b013e3181ebcc9c.
2. Richards, E.G., Zaveri, H.P., Wolf, V.L., Kang, S.L., Scott, D.A. (2011). Delineation of a less than 200 kb minimal deleted region for cardiac malformations on chromosome 7p22. Am J Med Genet A. *155A*(7), 1729–1734. doi: 10.1002/ajmg.a.34041.
3. Silversides, C.K., Lionel, A.C., Costain, G., Merico, D., Migita, O., Liu, B., Yuen, T., Rickaby, J., Thiruvahindrapuram, B., Marshall, C.R. (2012). Rare copy number variations in adults with tetralogy of Fallot implicate novel risk gene pathways. PLoS Genet. *8*(8), e1002843. doi: 10.1371/journal.pgen.1002843.
4. Rendu, J., Satre, V., Testard, H., Devillard, F., Vieville, G., Fauré, J., Amblard, F., Jouk, P-S. Coutton, C. (2014). 7p22.3 microdeletion disrupting SNX8 in a patient presenting with intellectual disability but no tetralogy of Fallot. Am J Med Genet A. *164A*(8), 2133–2135. doi: 10.1002/ajmg.a.36566.
5. Yu, A.C., Zambrano, R.M., Cristian, I., Price, S., Bernhard, B., Zucker, M., Venkateswaran, S., McGowan-Jordan, J., Armour, C.M. (2017). Variable developmental delays and characteristic facial features-A novel 7p22.3p22.2 microdeletion syndrome? Am J Med Genet A. *173*(6), 1593–1600. doi: 10.1002/ajmg.a.38241.
6. Mastromoro, G., Capalbo, A., Guido, C.A., Torres, B., Fabbretti, M., Traversa, A., Giancotti, A., Ventriglia, F., Bernardini, L., Spalice, A. et al. (2020). Small 7p22.3 microdeletion: Case report of Snx8 haploinsufficiency and neurological findings. Eur J Med Genet. *63*(4), 103772. doi: 10.1016/j.ejmg.2019.103772.
7. Skvortsova, L., Perfilyeva, A., Bespalova, K., Kuzovleva, Y., Kabysheva, N., Khamdiyeva, O. (2024). 7p22.3 microdeletion: A case study of a patient with congenital heart defect, neurodevelopmental delay and epilepsy. Orphanet J Rare Dis. *19*(1), 301–308. doi: 10.1186/s13023-024-03321-8.
8. Perez, G., Barber, G.P., Benet-Pages, A., Casper, J., Clawson, H., Diekhans, M., Fischer, C., Navarro Gonzalez, J., Hinrichs, A.S., Lee, C.M., et al. (2025). The UCSC genome browser database: 2025 update. Nucleic Acids Res. 53(D1), D1243–D1249. doi: 10.1093/nar/gkae974.
9. Richards, S., Aziz, N., Bale, S., Bick, D., Das, S., Gastier-Foster, J., Grody, W.W., Hegde, M., Lyon, E., Spector, E., et al. (2015). Standards and guidelines for the interpretation of sequence variants: a joint consensus recommendation of the American College of Medical Genetics and Genomics and the Association for Molecular Pathology. Genet Met 17(5), 405-424. doi: 10.1038/gim.2015.30.
