# Supplementary material for: A cardiovascular, craniofacial, and neurodevelopmental disorder caused by loss-of-function variants in the eIF3 complex component genes EIF3A and EIF3B
Source: Am J Hum Genet. 2025 Sep 30;112(11):2625–42. doi: 10.1016/j.ajhg.2025.09.008 (PMC12808965; doi:10.1016/j.ajhg.2025.09.008)
Supplement: Document S2. Article plus supplemental information [file mmc14.pdf]

A cardiovascular, craniofacial, and neurodevelopmental disorder caused by loss-of-function variants in the eIF3 complex component genes *EIF3A* and *EIF3B*

Authors

Esra Erkut, Cherith Somerville, Marci L. B. Schwartz, ..., Raymond H. Kim, Ian C. Scott, Rebekah K. Jobling

Correspondence

ian.scott@sickkids.ca (I.C.S.),
rebekah.jobling@sickkids.ca (R.K.J.)

Loss-of-function variants in *EIF3A* and *EIF3B* can lead to a variable multisystem congenital disorder, primarily involving cardiac anomalies, craniofacial differences, and mild neurodevelopmental features. These findings are supported by an international clinical cohort and zebrafish mutant models.

Erkut et al., 2025, The American Journal of Human Genetics *112*, 2625–2642

November 6, 2025 © 2025 The Authors. Published by Elsevier Inc. on behalf of American Society of Human Genetics.

https://doi.org/10.1016/j.ajhg.2025.09.008

A cardiovascular, craniofacial, and neurodevelopmental disorder caused by loss-of-function variants in the eIF3 complex component genes *EIF3A* and *EIF3B*

Esra Erkut,[1,2,29] Cherith Somerville,[3,29] Marci L.B. Schwartz,[3,4] Laura McDonald,[1] Qiliang Ding,[3] Olivia M. Moran,[3,4] Xin Chen,[3] Roozbeh Manshaei,[3] Anne-Sophie Riedijk,[1] Marie-Therese Schnürer,[1] Daniel C. Koboldt,[5] Stylianos E. Antonarakis,[6] Emma C. Bedoukian,[7] Xavier Blanc,[6] Laura K. Conlin,[7] Helen Cox,[8] Karin E.M. Diderich,[9] Bri Dingmann,[10] Christèle Dubourg,[11] Frances Elmslie,[12] Luis F. Escobar,[13] Rachel Gosselin,[14] Maria J. Guillen Sacoto,[15] Cynthia D. Haag,[16] Lisa Herzig,[10] Ramanand Jeeneea,[8] Priti Kenia,[17] Konstantinos Kolokotronis,[18] Anna M. Kopps,[19] Christin Kupper,[18] Hayley Lees,[20] Jacqueline Leonard,[7] Jonathan Levy,[21] Rebecca Littlejohn,[22] Demian Mayer,[19] Scott D. McLean,[22] Nikhil Pattani,[12] Laurence Perrin,[21] Véronique Pingault,[23] Chloé Quelin,[24] Emmanuelle Ranza,[6] Anita Rauch,[18] Sara L. Reichert,[7] Joana Rosmaninho-Salgado,[25] Cara Skraban,[7] Sérgio Sousa,[25] Melissa Stuebben,[22] Paolo Zanoni,[19,26] Raymond H. Kim,[3,4,27] Ian C. Scott,[1,2,*] and Rebekah K. Jobling[3,4,28,*]

Summary

Syndromic cardiac malformations can result in morbidity, yet their genetic etiology is only understood for a subset of individuals. Genome sequencing efforts in congenital anomaly cohorts may identify disease-associated variants in previously unrecognized genes. Through international matchmaking efforts, we identified eighteen individuals in total with *de novo* or loss-of-function variants in *EIF3A* ($n = 4$) or *EIF3B* ($n = 14$). The clinical phenotype varied but predominantly included cardiac defects, craniofacial dysmorphisms, mild developmental delays, and behavioral abnormalities. These genes encode core subunits of the eukaryotic initiation factor 3 (eIF3) complex, which plays a critical role in binding mRNA transcripts to the 40S ribosomal subunit during translation initiation. Both genes are highly constrained against loss of function, and animal models have demonstrated that disruptions in the eIF3 complex result in a range of developmental defects, including cardiovascular malformations. Additionally, *EIF3B* is located within the minimally overlapping region implicated in cardiac anomalies associated with 7p22.3 microdeletions. We sought to further study the role of these genes in syndromic congenital heart disease. To explore their functional impact, we generated zebrafish models with mutations in the orthologous *eif3s10* and *eif3ba* genes, which resulted in developmental abnormalities, including thin heart tubes, lack of craniofacial cartilage, and embryonic lethality. We propose that pathogenic variants in *EIF3A*, as well as pathogenic variants or microdeletions involving *EIF3B*, cause a distinct autosomal-dominant neurodevelopmental syndrome characterized by cardiovascular and craniofacial manifestations.

Introduction

Congenital heart disease (CHD) is common, affecting ~1% of live births, and is often associated with significant morbidity and mortality.[1,2] While the genetic basis of CHD is increasingly recognized, an identifiable genetic cause is found in only ~35% of affected individuals (aneuploidy, copy-number variant, or monogenic variant), with a higher diagnostic yield in individuals with extracardiac abnormalities (i.e., syndromic CHD).[3–6] Genome and

[1]Program in Developmental, Stem Cell & Cancer Biology, The Hospital for Sick Children, Toronto, ON, Canada; [2]Department of Molecular Genetics, University of Toronto, Toronto, ON, Canada; [3]Ted Rogers Centre for Heart Research, Cardiac Genome Clinic, The Hospital for Sick Children, Toronto, ON, Canada; [4]Division of Clinical and Metabolic Genetics, Department of Pediatrics, The Hospital for Sick Children, Toronto, ON, Canada; [5]The Steve and Cindy Rasmussen Institute for Genomic Medicine at Nationwide Children's Hospital, Columbus, OH, USA; [6]Medigenome, Swiss Institute of Genomic Medicine, Geneva, Switzerland; [7]Children's Hospital of Philadelphia, Philadelphia, PA, USA; [8]Birmingham Women's and Children's Hospital, Birmingham, UK; [9]Department of Clinical Genetics, Erasmus MC, Rotterdam, the Netherlands; [10]Seattle Children's Hospital, University of Washington, Seattle, WA, USA; [11]Service de Génétique Moléculaire et Génomique, CHU Pontchaillou, Rennes, France; [12]St George's University Hospitals NHS Foundation Trust, London, UK; [13]Medical Genetics, Peyton Manning Children's Hospital, Ascension Health, Indianapolis, IN, USA; [14]Division of Genetic and Genomic Medicine at Nationwide Children's Hospital, Columbus, OH, USA; [15]GeneDx LLC, Gaithersburg, MD, USA; [16]Medical Genetics, Ascension St. Vincent, Indianapolis, IN, USA; [17]Birmingham Women's and Children's NHS Trust, Birmingham, UK; [18]Institute of Medical Genetics, University of Zurich, Zurich, Switzerland; [19]Genetica AG, Zurich, Switzerland; [20]Exeter Genomics Laboratory, Royal Devon University Healthcare NHS Foundation Trust, Exeter, UK; [21]Genetics Department, AP-HP, Robert Debré University Hospital, Paris, France; [22]Baylor College of Medicine, Houston, TX, USA; [23]Service de Médecine Génomique des Maladies Rares, AP-HP, Hôpital Necker, Université Paris Cité, Paris, France; [24]Service de Génétique Clinique, CLAD Ouest, CHU Rennes, Rennes, France; [25]Medical Genetics Unit, Hospital Pediátrico, Centro Hospitalar e Universitário de Coimbra, Coimbra, Portugal; [26]Luzerner Kantonsspital, Lucerne, Switzerland; [27]Fred A. Litwin Family Centre in Genetic Medicine, University Health Network, Department of Medicine, University Health Network, Toronto, ON, Canada; [28]Genome Diagnostics, Department of Pediatric Laboratory Medicine, The Hospital for Sick Children, Toronto, ON, Canada
[29]These authors contributed equally
*Correspondence: ian.scott@sickkids.ca (I.C.S.), rebekah.jobling@sickkids.ca (R.K.J.)
https://doi.org/10.1016/j.ajhg.2025.09.008.
© 2025 The Authors. Published by Elsevier Inc. on behalf of American Society of Human Genetics.
This is an open access article under the CC BY license (http://creativecommons.org/licenses/by/4.0/).

exome sequencing continue to reveal disease-causing variants in genes associated with syndromic and nonsyndromic CHD, including those with well-established associations, as well as those for which evidence is still emerging. As part of a broader effort to sequence the genomes of individuals with CHD at the Hospital for Sick Children (SickKids), the Cardiac Genome Clinic initially identified two unrelated individuals with *de novo* loss-of-function (LoF) variants in genes encoding core subunits of the eukaryotic initiation factor 3 (eIF3) complex: *EIF3A* (MIM: 602039) and *EIF3B* (MIM: 603917). Through international matchmaking efforts, we assembled a cohort of eighteen individuals with *de novo* or LoF variants in *EIF3A* ($n = 4$) or *EIF3B* ($n = 14$).

EIF3A and *EIF3B* encode two of thirteen subunits of the eIF3 complex, the largest translation initiation factor complex, which plays a critical role in the initiation of protein synthesis.[7–9] The eIF3 complex binds to the 40S small ribosomal subunit, preventing its premature association with the 60S ribosomal subunit and facilitating mRNA transcript binding to the ribosome.[10] Recent evidence suggests that eIF3A and eIF3B can directly bind to mRNA and may provide transcript specificity during translation.[8] Both subunits function as scaffold proteins that are essential for proper assembly of the eIF3 complex, a role that is highly conserved across eukaryotic species.[11,12] eIF3a, the largest subunit of the eIF3 complex, is critical for the stabilization of the complex on the 40S ribosomal subunit[7,13] and can regulate the selective translation of specific mRNAs.[14,15] The eIF3b subunit is essential for anchoring the eIF3 complex to the small ribosomal subunit and facilitating the assembly of the translation initiation machinery.[16] *EIF3A* and *EIF3B* are ubiquitously expressed across tissues and developmental stages.[12,17] Additionally, their pLOEUF (putative LoF observed/expected upper fraction) scores in the Genome Aggregation Database (gnomAD), 0.18 for *EIF3A* and 0.11 for *EIF3B*, indicate a high intolerance to LoF variants for both genes.[18]

EIF3B is located within the boundary of microdeletions reported at chromosome region 7p22.3 in individuals with developmental delay, intellectual disability, craniofacial dysmorphisms, and CHD, particularly tetralogy of Fallot (TOF).[19–21] Notably, *EIF3B* is one of only two genes within the smallest region of overlap among individuals affected by CHD.[22–28] Regarding *EIF3A*, a missense variant, c.1145A>G (GenBank: NM_003750.4) (p.Tyr382Cys), was found to segregate with left-ventricular noncompaction (LVNC) in two related individuals.[29] Given the strong constraint against LoF in these genes and their observed associations with disease phenotypes, we investigated *EIF3A* and *EIF3B* variants as potential contributors to syndromic cardiac malformations.

In this study, we report the phenotype and genotype of fourteen unrelated individuals with heterozygous LoF and/or *de novo* heterozygous variants in *EIF3B*, as well as four unrelated individuals with *de novo* heterozygous LoF variants in *EIF3A*. These individuals present with a variable phenotype that includes CHD, craniofacial dysmorphisms, and mild neurodevelopmental abnormalities. We further validated the link between the loss of eIF3a or eIF3b and the development of CHD and craniofacial dysmorphism using zebrafish as an animal model. Zebrafish mutants exhibited severe developmental defects, including hypoplastic heart tubes, absent craniofacial cartilage, coloboma, reduced overall size, and embryonic lethality. Integrating the findings from our human cohort and zebrafish models, we propose that haploinsufficiency of either *EIF3A* or *EIF3B* causes an eIF3-related cardiovascular, craniofacial, and neurodevelopmental disorder.

Subjects, material, and methods

Subject recruitment

Two initial individuals were identified through the Cardiac Genome Clinic and subjected to a whole-genome short-read sequencing study at the Hospital for Sick Children (SickKids) and the Ted Rogers Centre for Heart Research.[30] The remaining sixteen individuals were identified via GeneDx, GeneMatcher,[31,32] and the Genomics England database.[33] Detailed clinical data for each proband were collected from collaborating institutions. Informed consent was obtained from all individuals and their families through the respective institution's research ethics board (REB)/institutional review board (IRB). This study was approved by the REB at the Hospital for Sick Children (SickKids), Toronto, ON, Canada (REB #1000053844).

Identification and evaluation of the variants

Variants in *EIF3B* and *EIF3A* were identified in probands using massively parallel sequencing (next-generation sequencing [NGS]) technologies, including exome and genome sequencing, in clinical diagnostic or research settings. Parental testing for the identified variant was performed when possible ($n = 17$). Candidates from Genomics England were identified by querying for pLoF variants using the Interactive Variant Analysis (IVA) tool within the Genomics England research environment.[33] Clinical collaboration requests were sent to providers of all rare disease cohort participants with Integrative Genomics Viewer-confirmed pLoF variants as of April 2024, inviting them to participate in the clinical series. To assess the presence of the variants in control populations, we used gnomAD (v.4.1.0).[18] All variants are described using the GenBank: NM_003750.4; NP_003741.1 MANE select transcript of *EIF3A* and the GenBank: NM_001037283.2; NP_001032360.1 MANE select transcript of *EIF3B*.[34]

Zebrafish husbandry

Adult TLAB zebrafish (*Danio rerio*) were maintained in accordance with the Canadian Council on Animal Care (CCAC) and the Hospital for Sick Children Animal

Services (LAS) guidelines. Embryos were raised in embryo medium at 28.5°C, as described in "The Zebrafish Book."[35] Stable transgenic lines were used to characterize defects in mutants, including *Tg(myl7:EGFP)*^{twu34}[36] and *Tg(gata1:dsRed)*^{sd2}.[37]

Zebrafish mutant generation and genotyping

The CRISPR-Cas9 system was used to target early exons in *eif3s10* and *eif3ba*. Guide RNAs (gRNAs) were designed using Benchling (www.benchling.com) and synthesized with the HiScribe T7 Quick High Yield RNA Synthesis Kit (NEB), followed by isopropanol/acetate precipitation.[38] The Cas9 protein was purified as described by Gagnon et al.,[39] with two modifications: cultures were induced with 0.3 mM IPTG (instead of auto-induction) and the dialysis buffer contained 10% glycerol. gRNAs and Cas9 were mixed in a 1:1 ratio (4 μM each) with a final concentration of 300 mM KCl to keep Cas9 in the solution[40] and then incubated at 37°C for 5 min before injection. 1 nL of the gRNA/Cas9 solution was injected into the yolk at the one-cell stage. Once the injected embryos reached adulthood, they were outcrossed to identify founders with germline mutations. Mutants carrying the desired allele were then outcrossed for at least three generations (F3) before being incrossed for phenotyping.

The *eif3s10* guide sequence is 5′-GAAGACCGAGACCGCTAAAG-3′. Two alleles were isolated for *eif3s10*: a −1 allele (hsc223) or a +5 A>G allele (hsc224), both located in exon 3 and leading to a premature stop codon early in exon 4 (Figure S7A). As both alleles exhibited the same phenotype (Figure S7B), allele hsc224 was used for the remainder of the study. Genotyping was performed using high-resolution melt (HRM) analysis,[41] with the following primers: *eif3s10* HRM F: 5′-TCGTCCGTGCTTACCTCAAAC-3′ and *eif3s10* HRM R: 5′-ATCCTCAATGTCCAGAACCATCT-3′. To confirm the specific insertion or deletion (indel) and generation of a premature stop codon, the region surrounding the indel was PCR amplified using the primers *eif3s10* seq F: 5′-GGAAGACAACCTCTCTCTCTGGTT-3′ and *eif3s10* seq R: 5′-TCGAGCAGCAAAATGAAGCTGAA-3′. Sanger sequencing was performed by the Centre for Applied Genomics (TCAG) at SickKids.

The *eif3ba* guide sequences are g1, 5′-GCCTTCGTTCAGTGATCCGG-3′ and g2, 5′-GGAGGCTGACGGCATCGACT-3′. One allele was isolated using guide 1 (g1): a +4 C>T in exon 1 (hsc225) (Figure S7A). A second allele was isolated using guide 2 (g2): a −4 deletion in exon 2 (hsc226) (Figure S7A). Both alleles resulted in a premature stop codon and exhibited the same phenotype (Figure S7B); therefore, the allele hsc225 was used for the remainder of the study. Genotyping was performed using HRM analysis with the following primers (for g1 and g2, respectively): *eif3ba* HRM 1F: 5′-AGGCCTCTCTCTCACCCTGAT-3′ and *eif3ba* HRM 1R: 5′-ATGGAGGCCGAAATGGAGTATG-3′, or *eif3ba* HRM 2F: 5′-CCGACCTGAGGCACATTATCAA-3′ and *eif3ba* HRM 2R: 5′-GATGTTCTCAGGGACAAACCG-3′. Indels were then confirmed by Sanger sequencing of PCR products surrounding the cut sites, using the following primers (g1 and g2, respectively): *eif3ba* seq g1F: 5′-CATGGTACGGTCCTTCTCTCG-3′ and *eif3ba* seq g1R: 5′-GGCATTAACTGTGAAACAGCGT-3′, or *eif3ba* seq g2F: 5′-GCAGCTGTTATGCTTTTGGCTT-3′ and *eif3ba* seq g2R: 5′-GCAGACCCAAACATACCCCTT-3′. Sequencing was performed by the TCAG at SickKids.

Imaging

Bright-field and fluorescent images and videos were captured using a Zeiss AXIO Zoom V16 microscope with ZEN software (Carl Zeiss AG). Image processing was performed in Fiji,[42] where images were cropped and the brightness/contrast were slightly adjusted when necessary. For heart rate analysis with PyHeart4Fish,[43] 500 frames were captured (exposure: 40 milliseconds, frame rate: 25 frames per second). Embryos were treated with 1-phenyl-2-thiourea (PTU) 1 day post-fertilization (dpf) to prevent the development of pigmentation, which would hinder video analysis. Still images of live embryos were obtained after anesthetizing them with tricaine and immobilizing them in 3% methyl cellulose.

Immunofluorescence

Immunofluorescence staining was performed as described by Rosenthal et al.,[44] with minor modifications. Briefly, embryos were fixed at 3 dpf in 4% paraformaldehyde (PFA) overnight, followed by washes in phosphate-buffered saline (PBS) + 0.1% Tween 20 (PBSTw) and dehydration through a methanol gradient. Embryos were stored in 100% methanol at −20°C until staining. Prior to staining, embryos were rehydrated (reverse methanol gradient) and bleached with a solution of 0.8% KOH, 0.9% H_2O_2, and 0.1% Tween 20 in sterile water until the eyes appeared clear.[45] Next, the embryos were permeabilized with ice-cold acetone and blocked with 10% normal goat serum + 5% bovine serum albumin in PBSTx (PBS with 1% Triton X-100), as described by Rosenthal et al.[44] The embryos were incubated overnight at 4°C with mouse anti-HuC/HuD monoclonal primary antibody (clone 16A11, #A-21271, Invitrogen) at a 1:500 dilution in blocking buffer. After PBSTx washes, goat anti-rabbit immunoglobulin (Ig)G (H+L) cross-adsorbed secondary antibody conjugated to Alexa Fluor 488 (#A-11008, Invitrogen) was added at a 1:200 dilution in blocking buffer and incubated overnight at 4°C. Following the final PBSTw washes, embryos were mounted in methyl cellulose for imaging and subsequently genotyped via HRM.

Alcian blue staining

Alcian blue staining for cartilage was performed as outlined in the referenced protocol (see web resources), with modifications to the bleaching procedure. The embryos were fixed at 5 dpf in 4% PFA overnight and then washed in PBSTw and dehydrated through a methanol gradient (50% MeOH/PBSTw and 100% MeOH). After

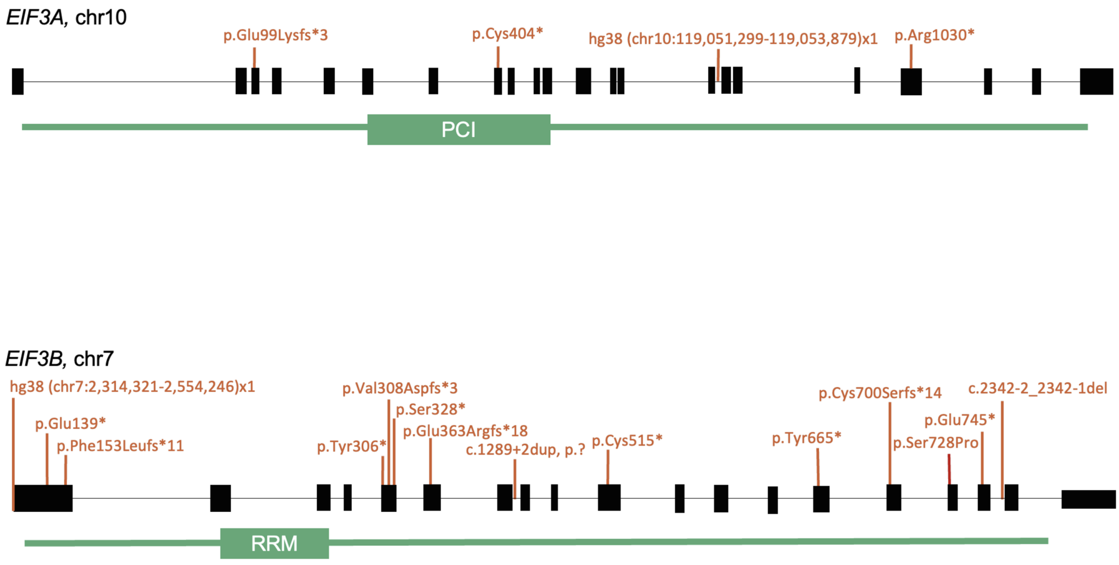

Figure 1. Schematic of *EIF3A* and *EIF3B* domains, highlighting the proteasome component domain in *EIF3A*, and the RNA recognition motif in *EIF3B*

Variants identified in the human cohort are shown in orange. The *EIF3B* full-gene deletion, hg38 (chr7:2314321–2554246)x1 also encompasses exon 1 of *SNX8*, *CHST12*, *GRIFIN*, *LFNG*, and several exons in the 3' end of *BRAT1*.

rehydration, the embryos were bleached in the dark as described for immunofluorescence and then rinsed with PBSTw before staining overnight at room temperature in an Alcian blue solution (1% concentrated hydrochloric acid, 70% ethanol, and 0.1% Alcian blue). The embryos were rinsed with acidic ethanol (70% EtOH + 5% HCl) and washed through a gradient of acidic ethanol/water (75%, 50%, and 25%) before final storage in 100% water. For imaging, the embryos were mounted in 80% glycerol-KOH, followed by genotyping via HRM.

Acridine orange staining

1 or 2 dpf embryos were manually dechorionated and then treated with 3 μg/mL acridine orange (in embryo medium) for 1 h in the dark at 28.5°C. Embryos were then washed twice for 5 min with embryo medium, anesthetized with tricaine, and mounted in 1% low melt agarose for imaging. Fiji[41] was used to identify fluorescent foci.

Results

Summary of clinical findings

Through the Cardiac Genome Clinic at SickKids,[30] GeneDx, GeneMatcher,[31,32] and Genomics England,[33] we studied fourteen unrelated individuals with heterozygous damaging *EIF3B* variants. This included ten individuals with LoF variants, one individual with a *de novo* microdeletion overlapping *EIF3B* and adjacent genes, one individual with a *de novo* splice acceptor variant, one individual with a *de novo* intronic variant, and one individual with a *de novo* missense variant (Figure 1). Additionally, four unrelated individuals with heterozygous *de novo* LoF variants in *EIF3A* were studied (Figure 1). Additional findings from genome or exome sequencing are detailed in Table S3.

EIF3B cohort

In the *EIF3B* cohort, eleven of fourteen individuals presented with CHD, including four individuals (probands #1, #2, #3, and #10) with TOF (Tables 1, 2, 3, and S1). One individual (proband #6) presented with a large perimembranous ventricular septal defect (VSD), small secundum atrial septal defect (ASD), pulmonary stenosis, and aortic dilation. Proband #8 presented with subaortic membrane stenosis, conoventricular VSD, aortic insufficiency, and an anomalous muscle bundle in the right ventricle, while proband #14 presented with pulmonary atresia with patent septum type 2. Other cardiac lesions included septal defects, bicuspid aortic valve (BAV), patent ductus arteriosus (PDA), and persistent left vena cava superior draining into the coronary sinus. Neurodevelopmental phenotypes were reported in eight individuals, including developmental delay, speech and language delay, intellectual disability, and mild or specific learning disabilities. Behavioral abnormalities, including attention-deficit hyperactivity disorder and autism spectrum disorder, were also noted. Facial differences were observed in eleven individuals (Tables 3 and S1; Figure 2). While features varied, several individuals exhibited differences in the eye region that overlapped with those previously reported in individuals with 7q22.3 microdeletions, including ptosis, arched eyebrows, downslanting palpebral fissures, hypertelorism, and epicanthal folds. Ptosis required intervention in proband #8. Cleft lip and palate were seen in four individuals in this study. One individual (proband #9) had a transverse terminal deficiency of the left hand (Figure S4; Table S1). Four probands were

Table 1. Summary of clinical characteristics in the *EIF3B* and *EIF3A* cohort and previously reported 7p22.3 microdeletion cases overlapping *EIF3B*

Clinical characteristics	*EIF3B* microdeletions (n = 10)[a]	*EIF3B* probands (n = 14)	*EIF3A* probands (n = 4)	Total (N = 28)
Developmental delay	7	6	1	14 (50%)
Intellectual disability/cognitive impairment	4	1	0	5 (18%)
Speech and language delay	5	5	2[b]	12 (43%)
Learning difficulties	N/A	2	1[c]	3 (11%)
Autism spectrum disorder	2	3	0	5 (18%)
Attention-deficit disorder	1	5	0	6 (21%)
Congenital heart defects	7	11	4	22 (79%)
Tetralogy of Fallot	3	4	2	9 (32%)
Craniofacial dysmorphisms	8	11	4	23 (82%)

N/A, not applicable.
[a]Published 7p22.3 microdeletions overlapping with *EIF3B* (Gallego et al.,[22] Richards et al.,[23] Silversides et al.,[24] Yu et al.,[26] and Skvortsova et al.[28]) (Table S2).
[b]One individual was previously reported to have speech and language delay but is currently within normal limits. The other individual was reported to have mild articulation problems.
[c]Query of a learning difficulty.

reported to have hearing loss and/or inner and middle ear malformations (Table S1).

EIF3A cohort

Within the *EIF3A* cohort, two individuals (probands #15 and #16) presented with TOF. A third individual (proband #17) had a perimembranous VSD, ASD, and patent foramen ovale. The fourth individual (proband #18) presented with VSD, right-sided aortic arch, and a vascular ring (Tables 1, 2, 3, and S1). One individual (proband #15) had a history of speech and language delays but is currently within normal limits. Proband #16 was suspected of having a learning difficulty, although all developmental milestones were met. Proband #17 exhibited mild articulation issues, and proband #18 was reported to have a developmental delay. Seizures were reported in one of these individuals. All four individuals exhibited nonspecific facial differences (Tables 3 and S1; Figure 2). Additional features observed included hearing loss, bilateral coloboma, and symphalangism of the left thumb in one individual (proband #16, Table S1).

Spectrum of the *EIF3B* LoF variants

Fourteen distinct *EIF3B* variants were identified (Tables 2 and S1; Figure 1). Ten were LoF sequence variants predicted to undergo nonsense-mediated decay (probands #1: Figure S1, family 1 #II-1; #3: Figure S1, family 3 #II-1; #5: Figure S1, family 5 #II-1; #6: Figure S1, family 6 #II-1; #8–#12: Figure S1, families 8–12 individuals #II-1; and #14: Figure S1, family 14 #II-1). The variant identified in proband #1,c.459del (GenBank: NM_001037283.2) (p.Phe153Leufs*11), was found to be maternally inherited, and the proband's mom presented with hip dysplasia. The variant identified in proband #14, c.918_919del (GenBank: NM_001037283.2) (p.Tyr306*), was also maternally inherited, and the mother was not reported to be clinically affected. Of the eight remaining truncating variants, seven were reported to be *de novo* when parental testing was available. The variant identified in proband #7 (Figure S1, family 7 #II-1), chr7:2314321–2554246 (GRCh38]) chr7:2353956–2593880 (GRCh37), is a *de novo* 240 kbp microdeletion at chromosome region 7p22.3 that overlaps the 5′ end of *SNX8* (MIM: 614905), *EIF3B*, *CHST12* (MIM: 610129), *GRIFIN* (MIM: 619187), and *LFNG* (MIM: 602576) and several exons in the 3′ end of *BRAT1* (MIM: 614506). The genes *SNX8*, *CHS12*, and *GRIFIN* do not have an established OMIM Morbid association, while *LFNG* and *BRAT1* are associated with autosomal-recessive phenotypes in OMIM (MIM: 609813, 618056, and 614498). Similar microdeletions overlapping *EIF3B* and additional adjacent genes have previously been reported in the literature in individuals with a variable phenotype that includes CHD, craniofacial malformations, and neurodevelopmental phenotypes[22–24,26,28] (Figure S3; Table S2). The *de novo* c.2342−2_2342−1del (GenBank: NM_001037283.2) (p.?) variant in proband #4 (Figure S1, family 4 #II-1) is located at the canonical acceptor splice site of the last coding exon (exon 18). RNA sequencing confirmed that this variant results in intron 17 retention and disruption of the protein starting from amino acid 782. However, it is not expected to undergo nonsense-mediated decay (Figure S5). The *de novo* variant in proband #13 (Figure S1, family 13 #II-1), c.1289+2dup (GenBank: NM_001037283.2) (p.?), is located within the splicing region of exon 7. The *in silico* splicing tools SpliceSiteFinder-Like,[46] MaxEnt,[47] and NNSPLICE[48] predict that it disrupts splicing by disrupting the canonical donor site at exon 7. RNA functional studies have not been performed for this variant. The *de novo* variant in proband #2 (Figure S1, family 2 #II-1), c.2182T>C (GenBank: NM_001037283.2)

Table 2. **Genetic information for probands with *de novo* or loss-of-function variants in *EIF3B* and *EIF3A***

Demographics and family information				Genetic information				
Proband	Sex	Age at last assessment	Family history	Gene	Variant	gnomAD frequency (v.4.1.0)	Inheritance pattern	Detection method
1	F	1 y, 1 mo	mother with hip dysplasia	*EIF3B*	c.459del (p.Phe153Leufs*11)	absent	maternal	GS
2	M	TOP at 22 6/7 weeks	none	*EIF3B*	c.2182T>C (p.Ser728Pro)	absent	*de novo*	ES
3	F	7 y, 8 mo	unknown; adopted	*EIF3B*	c.415G>T (p.Glu139*)	absent	unknown	ES
4	M	11 y	none	*EIF3B*	c.2342−2_2342−1del (p.?)	0.000063% (1 allele)	*de novo*	GS
5	F	9 y	none	*EIF3B*	c.923_924del (p.Val308Aspfs*3)	absent	*de novo*	ES
6	M	10 y, 8 mo	none	*EIF3B*	c.983C>G (p.Ser328*)	absent	*de novo*	GS
7	F	11 y, 11 mo	none	*EIF3B*	GRCh38/hg38 7p22.3 (chr7:2314321–2554246)x1 240 kbp	N/A	*de novo*	ES
8	M	9 y	none	*EIF3B*	c.1085dup (p.Glu363Argfs*18)	0.00012% (2 alleles)	*de novo*	ES
9	M	15 y	familial short stature	*EIF3B*	c.1545C>A (p.Cys515*)	absent	*de novo*	ES
10	M	9 mo	none	*EIF3B*	c.2233G>T (p.Glu745*)	absent	*de novo*	GS
11	F	2 mo	none	*EIF3B*	c.1995C>A (p.Tyr665*)	absent	*de novo*	ES
12	F	16 y, 4 mo	cleft palate	*EIF3B*	c.2097_2098dup (p.Cys700Serfs*14)	absent	unknown, not maternal	GS
13	M	3 mo	none	*EIF3B*	c.1289+2dup (p.?)	absent	*de novo*	ES panel
14	M	8 y	mother unaffected	*EIF3B*	c.918_919del (p.Tyr306*)	absent	maternal	GS
15	M	6 y	mother with ventricular septal defect	*EIF3A*	GRCh38/hg38 10q26.11 (chr10:119051299–119053879)x1 2.58 kbp	N/A	*de novo*	GS
16	F	6 y, 10 mo	family history of autism, ADHD, and heart problems; mother unaffected	*EIF3A*	c.295_296del (p.Glu99Lysfs*3)	absent	maternal	GS
17	F	1 y, 1 mo	mother had 1 early pregnancy loss	*EIF3A*	c.1209_1210del (p.Cys404*)	absent	*de novo*	ES
18	M	6 y	father with downslanting palpebral fissures	*EIF3A*	c.3088C>T (p.Arg1030*)	absent	*de novo*	ES

All *EIF3A* variants are mapped to GenBank: NM_003750.4; NP_003741.1, and all *EIF3B* variants are mapped to GenBank: NM_001037283.2; NP_001032360.1. ADHD, attention-deficit hyperactivity disorder; ES, exome sequencing; GS, genome sequencing; kbp, kilobase pairs; mo, months; N/A, not applicable; y, year(s); TOP, termination of pregnancy.

(p.Ser728Pro), is a missense change with *in silico* prediction scores of CADD: 25.8[49] and REVEL: 0.359.[50]

Spectrum of the *EIF3A* LoF variants

Four LoF variants were identified (Tables 2 and S1; Figure 1). A *de novo* 2.58 kbp intragenic deletion, chr10:119051299–119053879 (GRCh38) chr10:120810811–120813391 (GRCh37) at chromosome region 10q26.11, was identified in proband #15 (Figure S2, family 15 #II-1). The distal breakpoint of this deletion is located in intron 14 of *EIF3A*, while the proximal breakpoint extends 23 bp into the 5' end of exon 15, disrupting its canonical acceptor splice site. Sanger sequencing was used to confirm the deletion result and further define its exact genomic coordinates (Figure S6). Additionally, *de novo* nonsense variants, c.1209_1210del (GenBank: NM_003750.4) (p.Cys404*) and c.3088C>T (GenBank: NM_003750.4) (p.Arg1030*), were found in proband #17 (Figure S2, family 17 #II-1) and proband #18 (Figure S2, family 18 #II-1), respectively. A maternally inherited nonsense variant, c.295_296del (GenBank: NM_003750.4) (p.Glu99Lysfs*3), was also identified in another individual (proband #16; Figure S2, family 16

Table 3. Detailed clinical characteristics for probands with *de novo* or loss-of-function variants in *EIF3B* and *EIF3A*

Proband	Congenital heart defects	Craniofacial dysmorphisms	Behavioral and neurodevelopmental features	Seizure
1	TOF	none	none	–
2	TOF	large head, square face, microretrognathia	N/A	–
3	TOF	N/A	ADHD, aggression, DD, memory retention concerns	–
4	interatrial communication (spontaneously closed)	bilateral cleft lip and palate	delay in verbal language without intellectual disability, autism spectrum disorder	+
5	none	downslanting palpebral fissures, short neck, Noonan-like facies	ADHD, learning difficulties	–
6	pulmonary stenosis, ASD, VSD, aortic dilation	hypertelorism with telecanthus, arched eyebrows, eyelid ptosis, downslanting palpebral fissures, broad nasal root, posteriorly rotated ears, broad face, flattened and thin philtrum, Cupid's bow mouth	early DD, ADHD, autism spectrum disorder, dyslexia	–
7	none	cleft lip and palate	none; speech delay due to hearing loss	–
8	VSD, subaortic membrane stenosis, aortic insufficiency, anomalous muscle bundle of the right ventricle	ptosis	none	–
9	BAV	severe congenital ptosis OS > OD, dysplastic ears, mild malar hypoplasia, microcephaly	DD without cognitive impairment	–
10	TOF	bilateral cleft lip and palate, no teeth, large crus	N/A	–
11	VSD and ASD	microcephaly, dysmorphic features: broad forehead, temporal balding, arched eyebrows, broad nasal bridge, small nose, midface hypoplasia, flat philtrum, short neck	possible ADHD, mild language delay	–
12	none	none	mild learning disability	–
13	PDA, three small muscular VSD, perimembranous VSD, small ASD, persistent left vena cava superior draining in the coronary sinus	progressive microcephaly, high forehead with slightly prominent metopic suture, bilateral epicanthus, hypertelorism, prominent nasal tip, small mouth, retrognathia	none	–
14	pulmonary atresia with patent septum type 2	cleft lip and palate	intellectual disability, walked at 18 months, no language, hyperactivity, aggression, autism spectrum disorder	–
15	TOF	thin upper lip vermillion, retrognathia, epicanthus, anteverted nares, deeply set eyes	delayed speech and language now within normal limits	+
16	TOF with right-sided aortic arch	low-set posteriorly rotated ears, long philtrum, flat nasal bridge	met developmental milestones; 1:1 supervision at school for visual difficulties	–
17	VSD, ASD, PFO	thin upper vermillion, quite flat midface, downturned mouth corners	mild articulation problems	–
18	VSD, right-sided aortic arch, vascular ring	acrocephaly, downslanting palpebral fissures, epicanthal folds, telecanthus, deeply set eyes, maxillary hypoplasia, malar hypoplasia, micrognathia, triangular facies, asymmetry, tented lips, high arched palate, small flat teeth	DD	–

Please see Table S2 for the full table. ADHD, attention-deficit hyperactivity disorder; ASD, atrial septal defect; BAV, bicuspid aortic valve; DD, developmental delay; ECHO, echocardiogram; HC, head circumference; N/A, not applicable; NDD, neurodevelopmental delay; NGS, next-generations sequencing; OS > OD, more pronounced in the left eye (oculus sinister) compared to the right (oculus dexter); PDA, patent ductus arteriosus; PFO, patent foramen ovale; TOF, tetralogy of Fallot; VSD, ventricular septal defect; +, present; −, absent.

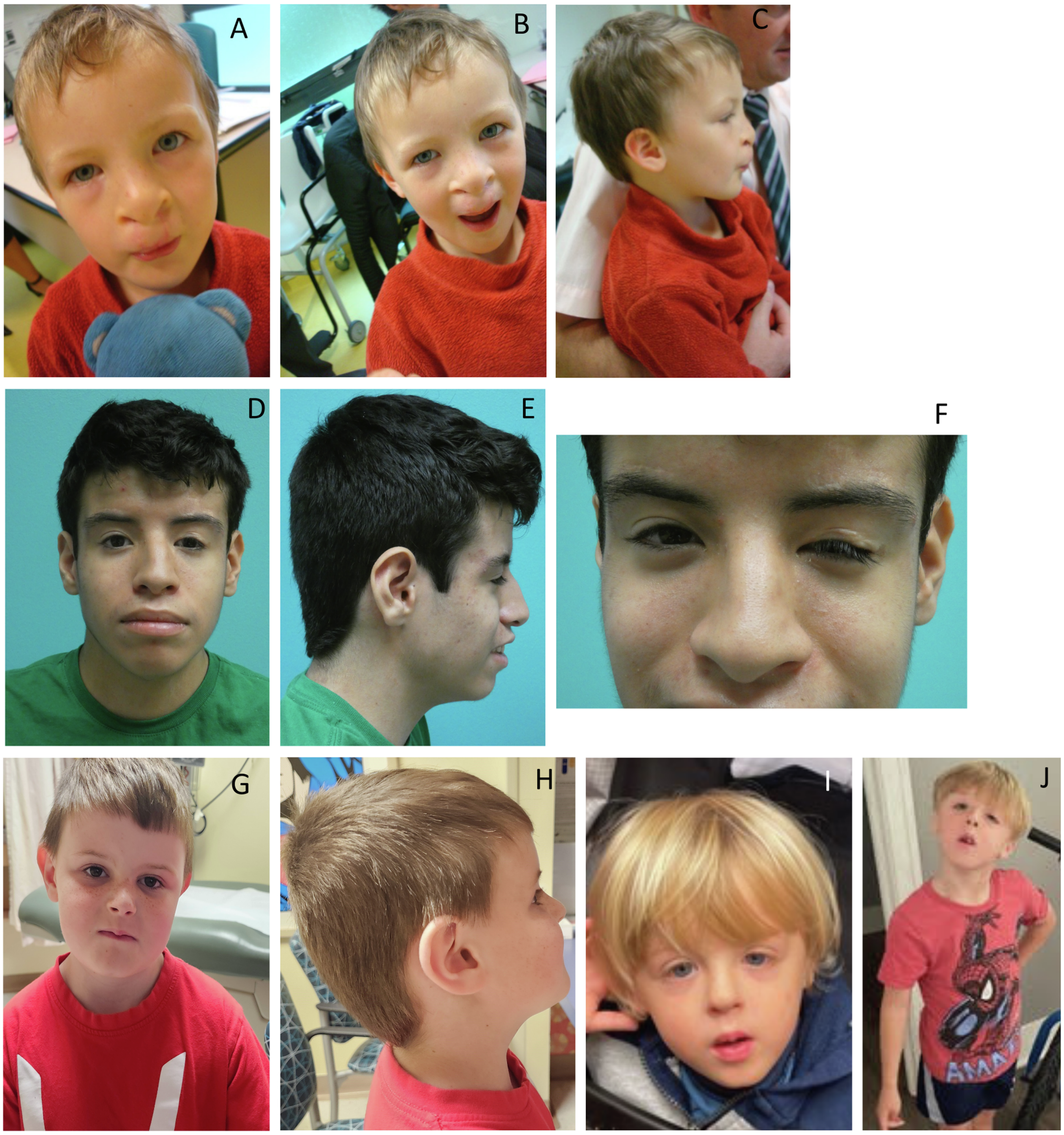

Figure 2. Photographs of probands with *de novo* variants in *EIF3B* or *EIF3A*
(A–C) Clinical images of proband #4, with a canonical acceptor splice site variant in *EIF3B*; (D–F) proband #9 with a loss-of-function variant in *EIF3B*; (G–H) proband #15, with a loss-of-function variant *EIF3A*; and (I and J) proband #18, with a loss-of-function variant in *EIF3A*.

#II-1), though the mother reportedly did not exhibit any related features.

Loss of orthologous *eif3s10* and *eif3ba* genes in zebrafish causes a spectrum of defects similar to those seen in the human cohort

To further investigate the link between *EIF3A* and *EIF3B* variants and the individuals' phenotypes described above, zebrafish mutant models were created using CRISPR-Cas9 to target early exons in the orthologous genes. *EIF3A* has a single ortholog, *eif3s10*, while *EIF3B* has two orthologs: *eif3ba* and *eif3bb*. The presence of two orthologs is common in zebrafish due to a whole-genome duplication event in the teleost lineage.[51] We chose to pursue *eif3ba* for this study, as targeting *eif3bb* did not cause a phenotype (data not shown), which may suggest divergence of its function. For *eif3s10* and *eif3ba*, the generated indels resulted in an early stop codon occurring upstream of or inside the essential protein domains (the proteasome component [PCI] domain in *eif3s10* or the RNA recognition motif [RRM] in *eif3ba*) (Figure S7A). Homozygous *eif3s10* and *eif3ba* mutants exhibited similar, fully penetrant phenotypes, while heterozygous loss of either gene caused no overt defects. Additionally, transheterozygote ($eif3ba^{+/-}$; $eif3s10^{+/-}$) mutants displayed no phenotype. In homozygous $eif3s10^{-/-}$ or $eif3ba^{-/-}$ mutants, minor

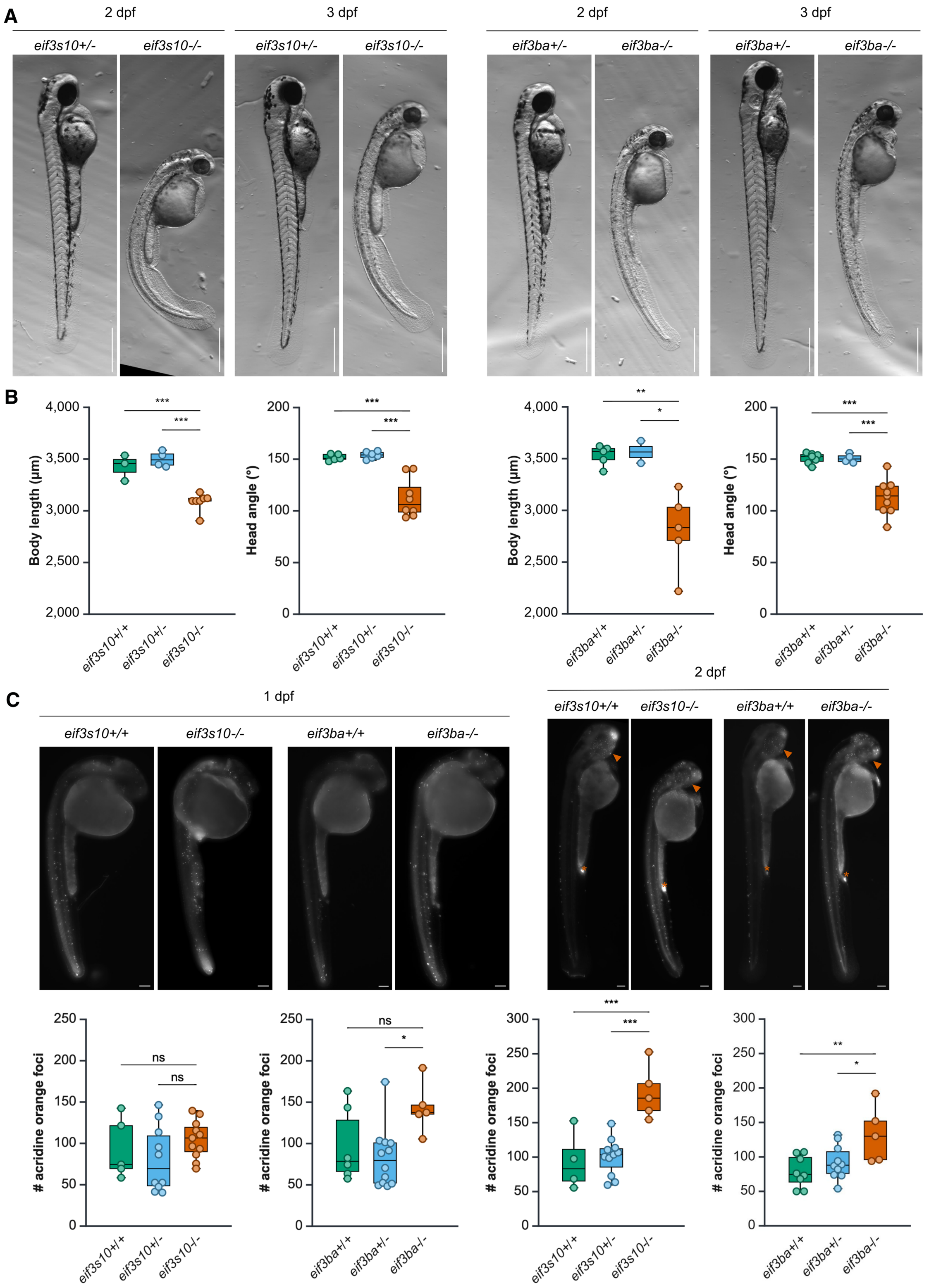

Figure 3. Loss of *eif3a* and *eif3b* in zebrafish causes a spectrum of defects similar to those observed in affected individuals
(A) Bright-field images of *eif3s10*$^{-/-}$ and *eif3ba*$^{-/-}$ embryos compared to heterozygous siblings at 2 and 3 dpf. Scale bars: 500 µm. Mutant embryos exhibit reduced size, delayed pigmentation, trunk curvature, and pericardial edema.
(B) Body length is significantly reduced at 3 dpf for both *eif3s10*$^{-/-}$ and *eif3ba*$^{-/-}$ mutant embryos. Additionally, head angle is significantly reduced at 3 dpf, indicative of a growth delay. Significance was determined by one-way ANOVA with Tukey multiple comparisons test; $^{*}p \leq 0.05$, $^{**}p \leq 0.01$, and $^{***}p \leq 0.001$. Each data point is one embryo, body length $n = 2$–7, and head angle $n = 4$–9.
(C) Acridine orange staining for apoptotic cells indicates a slight increase in apoptosis for *eif3ba*$^{-/-}$ embryos at 1 dpf, while there is no change for *eif3s10*$^{-/-}$ embryos. At 2 dpf, there is a significant increase in apoptosis for both *eif3s10*$^{-/-}$ and *eif3ba*$^{-/-}$ embryos when

(legend continued on next page)

yolk sac edema first became visible at approximately 26–28 h post-fertilization (hpf). By 30 hpf, mutants exhibited reduced eye size and worsening edema. The phenotype became extremely apparent by 2 days post-fertilization (dpf), characterized by severe pericardial edema, significantly reduced eye and head size, reduced pigmentation (particularly in the eye), and trunk curvature (Figure 3A). Embryo body length was also significantly reduced (Figure 3B). Many of the characteristics (small head, small eyes, short body length, and reduced pigmentation) point to an overall growth and developmental delay in mutant embryos. Additionally, mutants exhibited a significantly reduced head angle, which is routinely used to determine the age of embryos,[52] further providing evidence for a growth delay (Figure 3B). The reduced size may also be due to an increase in apoptosis in mutant embryos. To assess this, apoptotic cells were stained with the vital dye acridine orange at 1 and 2 dpf. At 1 dpf, there was a subtle increase in apoptosis for *eif3ba*$^{-/-}$ mutant embryos, while there was no significant change for *eif3s10*$^{-/-}$ mutant embryos (Figure 3C). However, by 2 dpf, there was a significant uptick in apoptosis compared to wild-type (WT) or heterozygous siblings (Figure 3C). This increase in cell death, combined with the overall growth delay, likely accounts for the reduced size and delayed morphological characteristics. Finally, mutant embryos showed no swimming activity and failed to exit their chorions spontaneously. While touch responsiveness was present at 24–30 hpf, it was lost by 3 dpf as the phenotype progressed. All mutants were uniformly dead by 5 dpf. Altogether, these findings demonstrate the essential roles of *eif3s10* and *eif3ba* during early development in zebrafish, aligning with the phenotypes observed in affected individuals.

eif3s10 and *eif3ba* LoF mutants have underdeveloped hearts and significantly impaired cardiac function

Given that many of the individuals exhibit TOF or other cardiac abnormalities, we closely investigated the cardiac phenotype in zebrafish *eif3s10* and *eif3ba* mutants. At 2 dpf, while WT and heterozygous siblings developed a looped, two-chambered heart, homozygous mutant embryos maintained a more linear heart tube (Figure 4A). By 3 dpf, these mutants exhibited hypoplastic heart tubes with little to no looping and severe pericardial edema. Their heartbeats were slow, weak, irregular, and insufficient for proper blood circulation (Videos S1, S2, and S3). To determine whether edema contributed to the phenotype, embryos were raised in 175 mM mannitol salts to balance osmolarity[53]; however, this treatment did not alleviate the stretched heart phenotype (Figure S7C), indicating a primary defect in heart development.

While 100% of the homozygous mutants exhibited some degree of cardiac abnormality at 3 dpf (and 100% were dead by 5 dpf), there was a range in severity. This was classified into four groups, ranging from “WT-like” (1) to most severe (4), based on the extent of the pericardial edema, size of chambers, and degree of looping (Figure 4B). Entire clutches of *eif3s10* or *eif3ba* embryos were scored at 3 dpf before genotyping, revealing that severe cardiac phenotypes (classes 3 and 4) were strongly associated with *eif3s10* or *eif3ba* loss compared to heterozygous or WT siblings. Heart rate and cardiac function of embryos were analyzed at 2 and 3 dpf via videomicroscopy using PyHeart4Fish[43] (Videos S4, S5, S6, S7, S8, S9, S10, and S11). Consistent with the phenotyping bins, these metrics were measured prior to genotyping. Heart rate was reduced in homozygous mutants compared to their heterozygous or WT siblings at both 2 and 3 dpf (Figure 4C). Additionally, mutants exhibited irregular heartbeats, with high arrhythmia scores (>0.7 indicates arrhythmia) at 2 dpf (Figure 4D). Heartbeat traces revealed further dysfunction, including simultaneous atrial and ventricular contractions (instead of sequential beating), prolonged pauses between beats, and missing atrial or ventricular contractions, resulting in disorganized traces (Figure 4E). These findings suggest defects in cardiac conduction system function. Altogether, these results demonstrate that *eif3s10* and *eif3ba* loss severely compromised both heart morphology and function in zebrafish mutants.

eif3s10 and *eif3ba* LoF mutants exhibit extracardiac abnormalities including reduced brain size, coloboma, and craniofacial cartilage defects

Beyond cardiac phenotypes, craniofacial dysmorphisms and mild neurodevelopmental abnormalities were among the most common characteristics observed in the human cohort. To investigate these features in our zebrafish mutants, we examined brain and craniofacial development. At 3 dpf, immunostaining for a pan-neuronal marker (HuC/HuD) revealed that homozygous mutants had significantly smaller brains compared to heterozygous or WT siblings (Figure 5A). Alcian blue staining showed a complete absence of craniofacial cartilage at 5 dpf in homozygous mutants, including the jaw and pharyngeal arches, retaining only minor cartilage deposition in the otic vesicle (Figure 5B). Mutant embryos also exhibited significantly smaller eyes when compared to their heterozygous or WT siblings, which is associated with microcephaly in zebrafish[54] (Figure 5C). Notably, mutants also exhibited coloboma (Figure 5C), a feature reported in one *EIF3A* individual (proband #16). Taken together, these findings demonstrate that loss of *eif3s10* or *eif3ba* in zebrafish results in a broad spectrum of

compared to their heterozygous or WT siblings. The orange asterisk (anal opening) and orange arrowhead (retina) indicate regions that have high levels of apoptosis during normal development; therefore, a high concentration of acridine orange is expected even in WT embryos. Significance was determined by one-way ANOVA with Tukey multiple comparisons test; $*p \leq 0.05$, $**p \leq 0.01$, and $***p \leq 0.001$. Scale bars: 100 μm. Each data point is one embryo, 1 dpf $n = 5$–12 embryos, and 2 dpf $n = 5$–11 embryos.

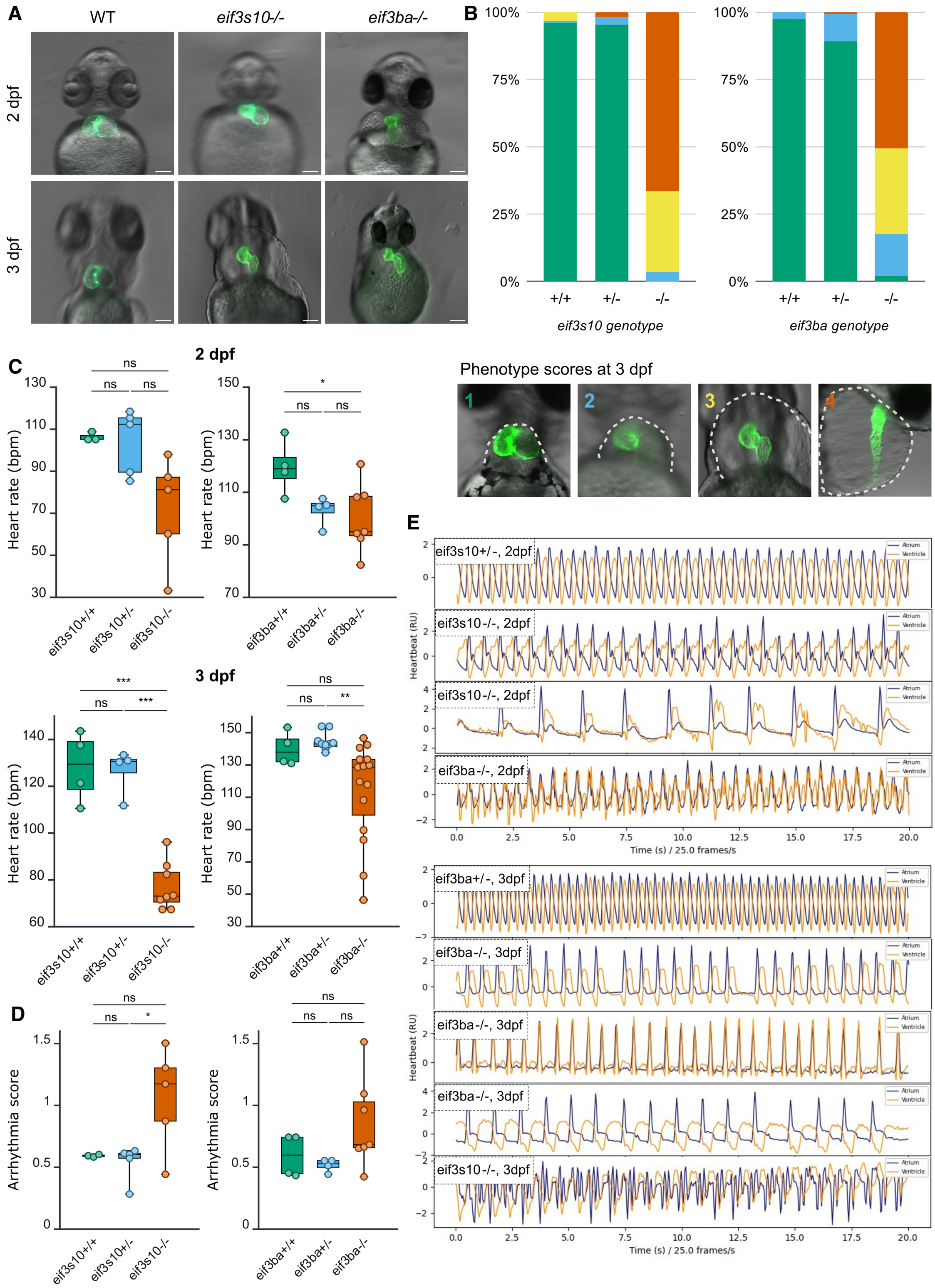

Figure 4. *eif3s10* and *eif3ba* loss-of-function mutants exhibit underdeveloped hearts and significantly impaired cardiac function

(A) At 2 dpf, homozygous mutants have stretched out hearts compared to WT. This worsens by 3 dpf, with hypoplastic heart tubes and severe pericardial edema. Scale bars: 100 μm.

(B) A greater proportion of homozygous mutants show "severe"-looking hearts at 3 dpf compared to heterozygous or WT siblings from the same clutch. Categories: 1 = WT-like, fully looped heart, no pericardial edema; 2 = heart is mostly looped, small pericardial edema; 3 = little to no looping, heart is stretched out, small chambers, large pericardial edema; and 4 = thin heart tube with no clear chambers or looping, severe pericardial edema that extends beyond the yolk (note: this image is lateral, the others are ventral). The gray dashed

(legend continued on next page)

developmental defects, consistent with the phenotypes observed in affected individuals.

Discussion

This study presents a cohort of individuals with heterozygous damaging variants in *EIF3A* and *EIF3B*, exhibiting a heterogeneous clinical phenotype primarily characterized by craniofacial abnormalities, CHD (most commonly TOF), and mild neurodevelopmental features. Among the fourteen damaging variants found in *EIF3B* and the four LoF variants found in *EIF3A*, thirteen were found to be *de novo*, two had unknown inheritance, and three were found to be maternally inherited (probands #1, #14, and #16). The mothers of these individuals were not reported to exhibit any relevant phenotypic features. Moreover, the variability observed in cardiac and craniofacial phenotypes is further suggestive of reduced penetrance and variable expressivity associated with clinically relevant variants in *EIF3A* and *EIF3B*. This clinical association is further supported by zebrafish models with null mutations in the orthologous genes, which largely match the range of phenotypes observed in affected individuals. Most variants in our cohort are LoF variants expected to result in nonsense-mediated decay, supporting haploinsufficiency as the mechanism of disease. The missense variant c.2182T>C (p.Ser728Pro) and the truncating variant c.2342−2_2342−1del in *EIF3B* do not directly impact known functional domains. More functional work is needed to determine how these variants impact protein function.

This study further supports haploinsufficiency of *EIF3B* as a contributing factor in 7p22.3 deletion syndrome, given that the gene is encompassed by the deleted region. Gallego et al.[22] described a male individual with a 2,880 kbp deletion containing *EIF3B*, presenting with VSD, developmental delay, cleft palate, failure to thrive, and hypotonia. His mother, who carried the same deletion, had mild intellectual disability, facial dysmorphisms, and velopharyngeal insufficiency. Richards et al.[23] reported an individual with a *de novo* ~400 kb deletion overlapping *MAD1L1* (MIM: 602686), *MRM2* (MIM: 606906), *NUDT1* (MIM: 600312), *SNX8*, and the first exon of *EIF3B*. This individual presented with TOF, language delay, facial dysmorphisms, and failure to thrive.[23] Similarly, Silversides et al.[24] reported a male individual with a 180 kb deletion spanning *SNX8*, *EIF3B*, and *CHST12*, who also had TOF. More recent studies have identified six additional individuals with 7p22.3 microdeletions associated with developmental delay, facial dysmorphisms, and CHDs, including TOF, ASD, double aortic arch, and VSD.[26,28] While *SNX8* has been considered as a candidate gene for the variable TOF and other CHDs in these individuals, neither *SNX8* nor other nearby genes within the deletion breakpoints are constrained against LoF (Table S4). Moreover, two microdeletions (<200 kbp) overlapping *SNX8* but not *EIF3B* have been reported in individuals without cardiac anomalies.[25,27] Overall, haploinsufficiency of *EIF3B* should be considered the primary cause for the cardiac phenotypes observed in individuals with this deletion and is also likely involved in the observed neurodevelopmental and craniofacial features, given the phenotypic overlap with our cohort of individuals with *EIF3A* or *EIF3B* pathogenic variants.

While some large-scale CHD genomic studies have not found damaging variants in *EIF3A* or *EIF3B,* one recent exome sequencing study reported high-confidence LoF *EIF3B* variants in two individuals with CHD (2/3,876) and none in control subjects (0/45,082),[55] further supporting the association between *EIF3B* and CHD. A previously reported missense variant in *EIF3A* (c.1145G>A [p.Tyr382Cys]) was identified in a family with LVNC and shown to affect cardiomyocyte proliferation, differentiation, and apoptosis.[29] While *in vitro* functional studies suggest that this variant may impact cardiomyocyte function, the association between these findings and the pathogenesis of LVNC has not been determined. The reported variant is present in 16 alleles in gnomAD v.4.1.1, and the phenotype differs from those associated with *EIF3A* LoF variants in our study. Therefore, the clinical significance of this variant remains unclear.

In humans, a different gene coding for a subunit in the eIF3 complex, *EIF3F* (MIM: 603914), is associated with autosomal-recessive intellectual developmental disorder 67 (MIM: 618295). This *EIF3F*-related syndrome is characterized by intellectual disability, developmental delay, behavioral abnormalities, hypertonia and/or hypotonia, hearing loss, and short stature.[56,57] Notably, delayed or absent speech development is a common feature within these reported cohorts. Additional findings include mild facial dysmorphisms, microcephaly, and ophthalmological manifestations.[56] Structural defects such as TOF, cleft lip and palate, and coloboma have

line in example images indicates the edge of pericardial edema. 3 clutches were scored per gene. *eif3s10* $n = 133$, 71, and 99 embryos. *eif3ba* $n = 88$, 169, and 112 embryos. Proportions of each phenotypic category per genotype were averaged prior to plotting.
(C) Mutant heart rate (beats per minute [bpm]), measured via PyHeart4Fish video analysis, was reduced compared to heterozygous and WT siblings. Significance was determined by one-way ANOVA with Tukey multiple comparisons test; $*p \le 0.05$, $**p \le 0.01$, and $***p \le 0.001$. Each data point is one embryo, and $n = 3$–15 embryos.
(D) Arrhythmia score at 2 dpf, measured by PyHeart4Fish, reveals irregular heartbeats in *eif3s10 and eif3ba* mutants. A lower score indicates more regular heartbeats, with 0.7 considered the cutoff for arrhythmia. Significance was determined by one-way ANOVA with Tukey multiple comparisons test; $*p \le 0.05$. Each data point is one embryo, and $n = 3$–7 embryos.
(E) Sample heartbeat traces at 2 and 3 dpf (measured by PyHeart4Fish) demonstrate irregular heartbeats for mutants vs. heterozygotes (top). Blue lines indicate atrial beats, while orange lines indicate ventricular beats.

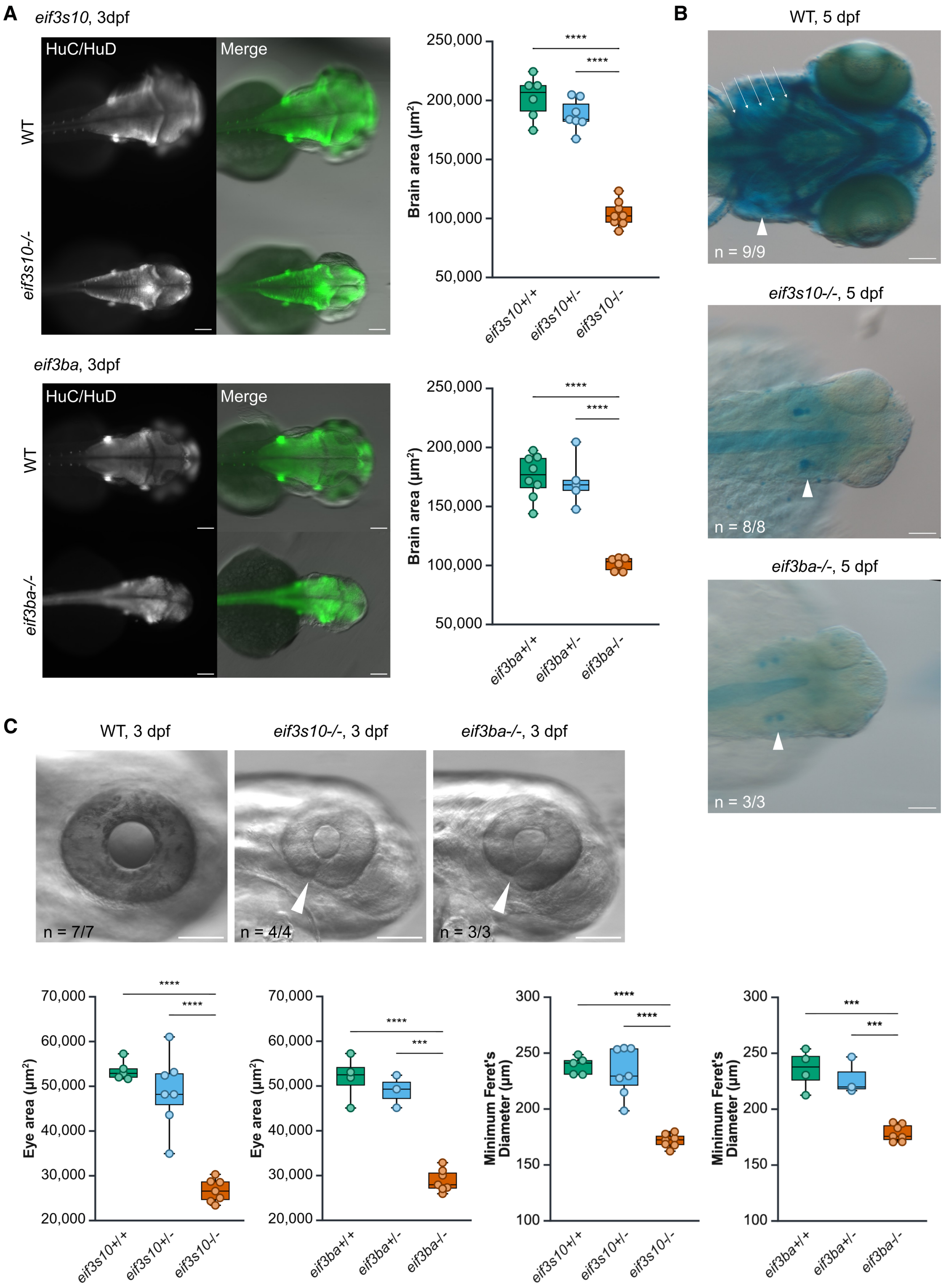

Figure 5. ***eif3s10* and *eif3ba* loss-of-function mutants exhibit extracardiac abnormalities including reduced brain size, coloboma, reduced eye size, and craniofacial cartilage defects**

(A) Immunofluorescent staining for a pan-neuronal marker (HuC/HuD) reveals reduced brain size in mutants compared to WT siblings at 3 dpf. Quantification revealed a significant decrease in brain area for both *eif3s10* and *eif3ba* mutants. Significance was determined by one-way ANOVA with Tukey multiple comparisons test; $*p \leq 0.05$, $**p \leq 0.01$, $***p \leq 0.001$, and $****p \leq 0.0001$. Each data point is one embryo, and $n = 5$–8 embryos. All scale bars: 100 µm.

(legend continued on next page)

also been observed in individuals with *EIF3F*-related disorder.[56] The presence of another disease-associated gene encoding an eIF3 subunit and the similar features observed in individuals with *EIF3F*-related disorder and those reported in our study provide further evidence of the critical role of the eIF3 complex and how disruption of a single subunit can contribute to disease. In addition to *EIF3F*, other subunits of the eIF complexes are associated with disease; for example, *EIF2B1* (MIM: 606686), *EIF2B2* (MIM: 606454), *EIF2B3* (MIM: 606273), *EIF2B4* (MIM: 606687), and *EIF2B5* (MIM: 603945) are associated with autosomal-recessive leukodystrophy phenotypes (MIM: 603896, 620312, 620313, 620314, and 620315), highlighting the vital role of proper eIF activity in postnatal brain function.

Our *eif3s10* and *eif3ba* zebrafish mutant models provide further evidence that disruption of human *EIF3A* or *EIF3B* can lead to a spectrum of developmental defects, including cardiovascular and craniofacial abnormalities. This study presents a comprehensive *eif3s10* zebrafish model, and while a previously published *eif3ba* mutant (generated via retroviral insertion) exists, we offer a more detailed characterization of the cardiac phenotype.[58] Our model largely recapitulates previous findings, including microcephaly, coloboma, delayed pigmentation, elongated hearts, and absent craniofacial cartilage. While additional mechanistic studies are required, it is possible that many of these defects stem, at least in part, from disruptions in neural crest cell development.[58] Neural crest cells contribute to multiple affected structures, including cardiac tissue (myocardium and outflow tract), craniofacial cartilage, melanocytes (pigmentation), and neurons.[59] Disruptions to cardiac neural crest cells have been implicated in the pathogenesis of several human cardiocraniofacial syndromes and cardiac outflow tract defects, including TOF.[5,6] We acknowledge that our zebrafish models are homozygous mutants, whereas the variants seen in the human cohort are heterozygous. It is a common occurrence in disease modeling for heterozygous variants that cause disease in humans to be tolerated in animal models. This is possibly due to the controlled lab setting, which prevents stressful environmental insults during development and thus hides any mild fitness defects. Additionally, in human individuals, other disease modifiers may be at play, which could further explain the phenotypic range. This phenomenon is exemplified for other CHD genes that are haploinsufficient in humans but require homozygous loss in animal models, including *TBX1* (MIM: 602054) (DiGeorge syndrome [DGS] [MIM: 188400]),[60,61] *TBX20* (MIM: 606061),[62,63] and *GATA4* (MIM: 600576).[64,65] Overall, our findings support the inclusion of *EIF3A* and *EIF3B* in genetic testing for CHD and other neurocristopathies.

Interestingly, despite their broad expression and essential cellular functions, *EIF3A* and *EIF3B* appear to cause a distinct developmental phenotype when disrupted. This may be due to heightened dosage sensitivity in the affected tissues or the presence of compensatory mechanisms in unaffected tissues. Several other "general" genes with essential cellular roles have been implicated in genetic disorders. For example, mutations in genes responsible for chromosomal structure maintenance cause Cornelia de Lange syndrome (CDLS [MIM: 122470]), which includes microcephaly, distinct facial features, intellectual deficits, and heart defects.[66] Similarly, mutations in *CHD7* (MIM: 608892) (encoding a chromatin remodeler) cause CHARGE syndrome (MIM: 214800),[67] while mutations in *PTPN11* (MIM: 176876) (encoding a protein tyrosine phosphatase involved in cell signaling) lead to Noonan syndrome (NS1 [MIM: 163950]).[68] Further investigation is required to uncover why certain organ systems, including neural crest-derived tissues, are more susceptible to disruptions in these broadly expressed genes.

Recent studies have highlighted the transcript selectivity of the eIF3 complex, suggesting that it plays a regulatory role at the level of translation. This may further explain why the loss of eIF3 function results in tissue-specific developmental defects. For example, Lee et al. profiled eIF3 binding targets in a human cell line and identified nearly 500 specific mRNA transcripts bound via their 5′ UTRs.[8] Notably, four of the thirteen eIF3 subunits, including eIF3a and eIF3b, directly crosslinked to RNA. These eIF3-bound transcripts are involved in key biological processes such as cell proliferation, differentiation, and apoptosis. A similar phenomenon was observed *in vivo,* where eIF3 was found to bind 5′ UTRs, specifically at a 20-nt UC-rich motif.[69] Gene Ontology (GO) analysis of these transcripts revealed enrichment for pathways related to cell migration, tissue morphogenesis, and differentiation, further supporting that eIF3 exerts some transcript selectivity during translation. Another eIF3 subunit, eIF3d, has been demonstrated to directly bind the 5′ cap of processed mRNA transcripts, providing additional evidence for transcript-specific regulation of translation by the eIF3 complex.[9] Moreover, eIF3 has been detected at the 3′ UTRs of highly translated transcripts during stem cell differentiation (i.e., at a time of drastic proteome changes), which may indicate another type of

(B) Dorsal view of Alcian blue-stained embryos reveals a lack of facial cartilage (including jaw and pharyngeal arches) in mutant embryos compared to WT at 5 dpf. White arrowheads indicate otic vesicles, and white arrows indicate pharyngeal arches. All scale bars: 100 μm.

(C) Mutant embryos have small eyes and coloboma at 3 dpf, with white arrowheads marking incomplete closure of the optic fissure. Quantification revealed a significant decrease in eye area and minimum Feret's diameter. All scale bars: 100 μm. Significance was determined by one-way ANOVA with Tukey multiple comparisons test; $*p \leq 0.05$, $**p \leq 0.01$, $***p \leq 0.001$, and $****p \leq 0.0001$. Each data point is one embryo (right eye measured only), and $n = 3–7$ embryos.

translational regulation by eIF3.[70] Altogether, the results show that transcript selectivity may contribute to the distinct phenotype observed in *EIF3A* and *EIF3B* haploinsufficiency. Further studies investigating the broader role of the eIF3 complex in translational regulation during development will help to better understand its pathological mechanism of action.

We have provided evidence for an eIF3-related cardiovascular, craniofacial, and neurodevelopmental disorder. This cohort of fourteen individuals with *EIF3B* variants and four with *EIF3A* variants depicts the variable phenotypic spectrum associated with *EIF3B*- and *EIF3A*-deficiency. Our targeted zebrafish models recapitulate key phenotypic features observed in affected individuals, further providing evidence that loss of *EIF3A* or *EIF3B* contributes to congenital anomalies. These findings underscore the importance of evaluating *EIF3A* and *EIF3B* in CHD cohorts. Expanding investigations into other clinical populations would help to better characterize the full phenotypic spectrum of *EIF3A* and *EIF3B* haploinsufficiency.

Data and code availability

There are restrictions to the availability of genome sequencing data due to privacy considerations, and institution-specific regulatory requirements. Anonymized data may be available upon reasonable request. Requests should be directed to and will be fulfilled by the lead contacts Dr. Rebekah Jobling (rebekah.jobling@sickkids.ca) and Dr. Ian Scott (ian.scott@sickkids.ca).

Acknowledgments

This research was made possible through access to data in the National Genomic Research Library, which is managed by Genomics England Limited (a wholly owned company of the Department of Health and Social Care). The National Genomic Research Library holds data provided by patients and collected by the NHS as part of their care and data collected as part of their participation in research. The National Genomic Research Library is funded by the National Institute for Health Research and NHS England. The Wellcome Trust, Cancer Research UK, and the Medical Research Council have also funded research infrastructure. We thank all families and their clinicians for partaking in this study. Zebrafish studies were supported by Canadian Institutes of Health Research (CIHR) funding (CIHR PJT 178155) and the Azrieli PCHP Catalyst Program (SickKids/Ste. Justine) provided to I.C.S. E.E. was supported by a CIHR Vanier Scholarship and a SickKids Restracomp Scholarship. We thank the zebrafish technicians at SickKids for animal husbandry. Thank you to Nathan Stutt for assistance with videomicroscopy of zebrafish hearts and Simon Monis for troubleshooting the PyHeart4Fish software. We thank the following labs for sharing reagents: Dr. James Ellis, Dr. Brian Ciruna, Dr. Madeline Hayes, and Dr. James Dowling. Boxplots in Figures 3, 4, and 5 were created with BioRender. Research reported in this publication was supported by the Ted Rogers Centre for Heart Research (R.K.J.), the Plan France Médecine Génomique 2025 (V.P., C.Q., and C.D.), the Nationwide Foundation Innovation Fund (D.C.K.), the ChildCare Foundation (S.E.A.), and the University of Zurich Clinical Research Priority Program (A.R.).

Author contributions

E.E. and C. Somerville wrote the paper, with input from all authors. I.C.S. and R.K.J. provided supervision. L.M. generated the zebrafish CRISPR mutants. I.C.S. and E.E. devised the zebrafish phenotyping experiments, and E.E. performed and analyzed the experiments. A.-S.R. and M.-T.S. assisted with zebrafish experiments. C. Somerville, Q.D., X.C., and R.M. were involved in genome analysis for probands #1 and #15. O.M. M. and R.K.J. provided genetic consultation for probands #1 and #15. M.L.B.S., X.C., O.M.M., R.H.K., I.C.S., R.K.J., and S. E.A. provided valuable direction in the development of the manuscript. All other authors contributed clinical/genetic analysis and provided clinical and genotypic data.

Declaration of interests

S.E.A. and E.R. are cofounders of Medigenome, Swiss Institute of Genomic Medicine. M.J.G.S. is an employee and may own stock in GeneDx.

Supplemental information

Supplemental information can be found online at https://doi.org/10.1016/j.ajhg.2025.09.008.

Web resources

CADD, https://cadd.gs.washington.edu/
ClinVar, https://www.ncbi.nlm.nih.gov/clinvar/
Ensembl, https://useast.ensembl.org/index.html
GenBank, https://www.ncbi.nlm.nih.gov/genbank
GeneMatcher, https://genematcher.org/
Gene Ontology, https://geneontology.org/
Genome Aggregation Database, https://gnomad.broadinstitute.org/
Genomics England, https://www.genomicsengland.co.uk/
OMIM, https://omim.org/
SDB Protocol: Staining and observation of cartilaginous structures in zebrafish complete larvae, https://www.sdbonline.org/sites/2008ShortCourse/Zebrafish_AlcianBlue.pdf
UCSC Genome Browser, https://genome.ucsc.edu/
UniProt, https://www.uniprot.org/

Received: April 7, 2025
Accepted: September 9, 2025
Published: September 30, 2025

References

1. Fahed, A.C., and Nemer, G.M. (2012). In Genetic causes of syndromic and non-syndromic congenital heart disease, N.D. Cooper and J.M. Chen, eds. (IntechOpen). https://doi.org/10.5772/48477.
2. Shen, L., Li, J., Zhang, H., and Zhao, Y. (2024). Global, regional and national burdens of reproduction-related congenital birth defects, 1990-2019. Front. Public Health *12*, 1328282. https://doi.org/10.3389/fpubh.2024.1328282.
3. Yasuhara, J., and Garg, V. (2021). Genetics of congenital heart disease: A narrative review of recent advances and clinical implications. Transl. Pediatr. *10*, 2366–2386. https://doi.org/10.21037/tp-21-297.

4. Simmons, M.A., and Brueckner, M. (2017). The genetics of congenital heart disease, understanding and improving long-term outcomes in congenital heart disease: A review for the general cardiologist and primary care physician. Curr. Opin. Pediatr. *29*, 520–528. https://doi.org/10.1097/MOP.0000000000000538.
5. Keyte, A., and Hutson, M.R. (2012). The neural crest in cardiac congenital anomalies. Differentiation. *84*, 25–40. https://doi.org/10.1016/j.diff.2012.04.005.
6. Dyer, L.A., and Rugonyi, S. (2021). Fetal blood flow and genetic mutations in conotruncal congenital heart disease. J. Cardiovasc. Dev. Dis. *8*, 90. https://doi.org/10.3390/jcdd8080090.
7. Masutani, M., Sonenberg, N., Yokoyama, S., and Imataka, H. (2007). Reconstitution reveals the functional core of mammalian eIF3. EMBO J. *26*, 3373–3383. https://doi.org/10.1038/sj.emboj.7601765.
8. Lee, A.S.Y., Kranzusch, P.J., and Cate, J.H.D. (2015). eIF3 targets cell-proliferation messenger RNAs for translational activation or repression. Nature *522*, 111–114. https://doi.org/10.1038/nature14267.
9. Lee, A.S., Kranzusch, P.J., Doudna, J.A., and Cate, J.H.D. (2016). eIF3d is an mRNA cap-binding protein that is required for specialized translation initiation. Nature *536*, 96–99. https://doi.org/10.1038/nature18954.
10. Kolupaeva, V.G., Unbehaun, A., Lomakin, I.B., Hellen, C.U.T., and Pestova, T.V. (2005). Binding of eukaryotic initiation factor 3 to ribosomal 40S subunits and its role in ribosomal dissociation and anti-association. RNA *11*, 470–486. https://doi.org/10.1261/rna.7215305.
11. Asano, K., Kinzy, T.G., Merrick, W.C., and Hershey, J.W. (1997). Conservation and diversity of eukaryotic translation initiation factor eIF3. J. Biol. Chem. *272*, 1101–1109. https://doi.org/10.1074/jbc.272.2.1101.
12. Wagner, S., Herrmannová, A., Šikrová, D., and Valášek, L.S. (2016). Human eIF3b and eIF3a serve as the nucleation core for the assembly of eIF3 into two interconnected modules: The yeast-like core and the octamer. Nucleic Acids Res. *44*, 10772–10788. https://doi.org/10.1093/nar/gkw972.
13. Aitken, C.E., Beznosková, P., Vlčkova, V., Chiu, W.-L., Zhou, F., Valášek, L.S., Hinnebusch, A.G., and Lorsch, J.R. (2016). Eukaryotic translation initiation factor 3 plays distinct roles at the mRNA entry and exit channels of the ribosomal preinitiation complex. eLife *5*, e20934. https://doi.org/10.7554/eLife.20934.
14. Baugh, J.M., and Pilipenko, E.V. (2004). 20S proteasome differentially alters translation of different mRNAs via the cleavage of eIF4F and eIF3. Mol. Cell *16*, 575–586. https://doi.org/10.1016/j.molcel.2004.10.017.
15. Yin, J.Y., Dong, Z.Z., Liu, R.Y., Chen, J., Liu, Z.Q., and Zhang, J.T. (2013). Translational regulation of RPA2 via internal ribosomal entry site and by eIF3a. Carcinogenesis *34*, 1224–1231. https://doi.org/10.1093/carcin/bgt052.
16. Erzberger, J.P., Stengel, F., Pellarin, R., Zhang, S., Schaefer, T., Aylett, C.H.S., Cimermančič, P., Boehringer, D., Sali, A., Aebersold, R., and Ban, N. (2014). Molecular architecture of the 40S·eIF1·eIF3 translation initiation complex. Cell *158*, 1123–1135. https://doi.org/10.1016/j.cell.2014.07.044.
17. Saletta, F., Suryo Rahmanto, Y., and Richardson, D.R. (2010). The translational regulator eIF3a: The tricky eIF3 subunit. Biochim. Biophys. Acta *1806*, 275–286. https://doi.org/10.1016/j.bbcan.2010.07.005.
18. Karczewski, K.J., Francioli, L.C., Tiao, G., Cummings, B.B., Alföldi, J., Wang, Q., Collins, R.L., Laricchia, K.M., Ganna, A., Birnbaum, D.P., et al. (2020). The mutational constraint spectrum quantified from variation in 141,456 humans. Nature *581*, 434–443. https://doi.org/10.1038/s41586-020-2308-7.
19. Speleman, F., Craen, M., and Leroy, J. (1989). De novo terminal deletion 7p22.1–pter in a child without craniosynostosis. J. Med. Genet. *26*, 528–532. https://doi.org/10.1136/jmg.26.8.528.
20. Chotai, K.A., Brueton, L.A., van Herwerden, L., Garrett, C., Hinkel, G.K., Schinzel, A., Mueller, R.F., Speleman, F., and Winter, R.M. (1994). Six cases of 7p deletion: Clinical, cytogenetic, and molecular studies. Am. J. Med. Genet. *51*, 270–276. https://doi.org/10.1002/ajmg.1320510320.
21. Grebe, T.A., Stevens, M.A., Byrne-Essif, K., and Cassidy, S.B. (1992). 7p deletion syndrome: An adult with mild manifestations. Am. J. Med. Genet. *44*, 18–23. https://doi.org/10.1002/ajmg.1320440106.
22. Gallego, C.J., Grant, J., Mikhail, F.M., Barger, C., and Robin, N.H. (2010). Use of array comparative genome hybridization in orofacial clefting. J. Craniofac. Surg. *21*, 1591–1594. https://doi.org/10.1097/SCS.0b013e3181ebcc9c.
23. Richards, E.G., Zaveri, H.P., Wolf, V.L., Kang, S.H.L., and Scott, D.A. (2011). Delineation of a less than 200 kb minimal deleted region for cardiac malformations on chromosome 7p22. Am. J. Med. Genet. *155A*, 1729–1734. https://doi.org/10.1002/ajmg.a.34041.
24. Silversides, C.K., Lionel, A.C., Costain, G., Merico, D., Migita, O., Liu, B., Yuen, T., Rickaby, J., Thiruvahindrapuram, B., Marshall, C.R., et al. (2012). Rare copy number variations in adults with tetralogy of Fallot implicate novel risk gene pathways. PLoS Genet. *8*, e1002843. https://doi.org/10.1371/journal.pgen.1002843.
25. Rendu, J., Satre, V., Testard, H., Devillard, F., Vieville, G., Fauré, J., Amblard, F., Jouk, P.-S., and Coutton, C. (2014). 7p22.3 microdeletion disrupting SNX8 in a patient presenting with intellectual disability but no tetralogy of Fallot. Am. J. Med. Genet. *164A*, 2133–2135. https://doi.org/10.1002/ajmg.a.36566.
26. Yu, A.C., Zambrano, R.M., Cristian, I., Price, S., Bernhard, B., Zucker, M., Venkateswaran, S., McGowan-Jordan, J., and Armour, C.M. (2017). Variable developmental delays and characteristic facial features-A novel 7p22.3p22.2 microdeletion syndrome? Am. J. Med. Genet. *173*, 1593–1600. https://doi.org/10.1002/ajmg.a.38241.
27. Mastromoro, G., Capalbo, A., Guido, C.A., Torres, B., Fabbretti, M., Traversa, A., Giancotti, A., Ventriglia, F., Bernardini, L., Spalice, A., and Pizzuti, A. (2020). Small 7p22.3 microdeletion: Case report of Snx8 haploinsufficiency and neurological findings. Eur. J. Med. Genet. *63*, 103772. https://doi.org/10.1016/j.ejmg.2019.103772.
28. Skvortsova, L., Perfilyeva, A., Bespalova, K., Kuzovleva, Y., Kabysheva, N., and Khamdiyeva, O. (2024). 7p22.3 microdeletion: A case study of a patient with congenital heart defect, neurodevelopmental delay and epilepsy. Orphanet J. Rare Dis. *19*, 301–308. https://doi.org/10.1186/s13023-024-03321-8.
29. Ge, M., Bai, X., Liu, A., Liu, L., Tian, J., and Lu, T. (2021). An eIF3a gene mutation dysregulates myocardium growth with left ventricular noncompaction via the p-ERK1/2 pathway. Genes Dis. *8*, 545–554. https://doi.org/10.1016/j.gendis.2020.02.003.

30. Reuter, M.S., Chaturvedi, R.R., Liston, E., Manshaei, R., Aul, R.B., Bowdin, S., Cohn, I., Curtis, M., Dhir, P., Hayeems, R.Z., et al. (2020). The cardiac genome clinic: Implementing genome sequencing in pediatric heart disease. Genet. Med. *22*, 1015–1024. https://doi.org/10.1038/s41436-020-0757-x.
31. Sobreira, N., Schiettecatte, F., Valle, D., and Hamosh, A. (2015). GeneMatcher: A matching tool for connecting investigators with an interest in the same gene. Hum. Mutat. *36*, 928–930. https://doi.org/10.1002/humu.22844.
32. Hamosh, A., Wohler, E., Martin, R., Griffith, S., Rodrigues, E.d.S., Antonescu, C., Doheny, K.F., Valle, D., and Sobreira, N. (2022). The impact of GeneMatcher on international data sharing and collaboration. Hum. Mutat. *43*, 668–673. https://doi.org/10.1002/humu.24350.
33. The National Genomic Research Library v5.1, Genomics England. doi: https://doi.org/10.6084/m9.figshare.4530893.v7.
34. Cunningham, F., Allen, J.E., Allen, J., Alvarez-Jarreta, J., Amode, M.R., Armean, I.M., Austine-Orimoloye, O., Azov, A.G., Barnes, I., Bennett, R., et al. (2022). Ensembl 2022. Nucleic Acids Res. *50*, D988–D995. https://doi.org/10.1093/nar/gkab1049.
35. Westerfield, M. (2000). The Zebrafish Book. A Guide for the Laboratory Use of Zebrafish (Danio rerio), 4th ed. (Univ. of Oregon Press).
36. Huang, C.J., Tu, C.T., Hsiao, C.D., Hsieh, F.J., and Tsai, H.J. (2003). Germ-line transmission of a myocardium-specific GFP transgene reveals critical regulatory elements in the cardiac myosin light chain 2 promoter of zebrafish. Dev. Dyn. *228*, 30–40. https://doi.org/10.1002/dvdy.10356.
37. Traver, D., Paw, B.H., Poss, K.D., Penberthy, W.T., Lin, S., and Zon, L.I. (2003). Transplantation and in vivo imaging of multilineage engraftment in zebrafish bloodless mutants. Nat. Immunol. *4*, 1238–1246. https://doi.org/10.1038/ni1007.
38. Varshney, G.K., Pei, W., LaFave, M.C., Idol, J., Xu, L., Gallardo, V., Carrington, B., Bishop, K., Jones, M., Li, M., et al. (2015). High-throughput gene targeting and phenotyping in zebrafish using CRISPR/Cas9. Genome Res. *25*, 1030–1042. https://doi.org/10.1101/gr.186379.114.
39. Gagnon, J.A., Valen, E., Thyme, S.B., Huang, P., Akhmetova, L., Pauli, A., Montague, T.G., Zimmerman, S., Richter, C., and Schier, A.F. (2014). Efficient mutagenesis by Cas9 protein-mediated oligonucleotide insertion and large-scale assessment of single-guide RNAs. PLoS One *9*, e98186. https://doi.org/10.1371/journal.pone.0098186.
40. Burger, A., Lindsay, H., Felker, A., Hess, C., Anders, C., Chiavacci, E., Zaugg, J., Weber, L.M., Catena, R., Jinek, M., et al. (2016). Maximizing mutagenesis with solubilized CRISPR-Cas9 ribonucleoprotein complexes. Development *143*, 2025–2037. https://doi.org/10.1242/dev.134809.
41. Parant, J.M., George, S.A., Pryor, R., Wittwer, C.T., and Yost, H.J. (2009). A rapid and efficient method of genotyping zebrafish mutants. Dev. Dyn. *238*, 3168–3174. https://doi.org/10.1002/dvdy.22143.
42. Schindelin, J., Arganda-Carreras, I., Frise, E., Kaynig, V., Longair, M., Pietzsch, T., Preibisch, S., Rueden, C., Saalfeld, S., Schmid, B., et al. (2012). Fiji: An open-source platform for biological-image analysis. Nat. Methods *9*, 676–682. https://doi.org/10.1038/nmeth.2019.
43. Vedder, V.L., Reinberger, T., Haider, S.M.I., Eichelmann, L., Odenthal, N., Abdelilah-Seyfried, S., Aherrahrou, Z., Breuer, M., and Erdmann, J. (2023). pyHeart4Fish: Chamber-specific heart phenotype quantification of zebrafish in high-content screens. Front. Cell Dev. Biol. *11*, 1143852. https://doi.org/10.3389/fcell.2023.1143852.
44. Rosenthal, S.M., Misra, T., Abdouni, H., Branon, T.C., Ting, A.Y., Scott, I.C., and Gingras, A.C. (2021). A toolbox for efficient proximity-dependent biotinylation in zebrafish embryos. Mol. Cell. Proteomics *20*, 100128. https://doi.org/10.1016/j.mcpro.2021.100128.
45. Huang, M., Akerberg, A.A., Zhang, X., Yoon, H., Joshi, S., Hallinan, C., Nguyen, C., Pu, W.T., Haigis, M.C., Burns, C.G., and Burns, C.E. (2022). Intrinsic myocardial defects underlie an rbfox-deficient zebrafish model of hypoplastic left heart syndrome. Nat. Commun. *13*, 5877. https://doi.org/10.1038/s41467-022-32982-x.
46. Leman, R., Gaildrat, P., Le Gac, G., Ka, C., Fichou, Y., Audrezet, M.P., Caux-Moncoutier, V., Caputo, S.M., Boutry-Kryza, N., Léone, M., et al. (2018). Novel diagnostic tool for prediction of variant spliceogenicity derived from a set of 395 combined in silico/in vitro studies: An international collaborative effort. Nucleic Acids Res. *46*, 7913–7923. https://doi.org/10.1093/nar/gky372.
47. Yeo, G., and Burge, C.B. (2004). Maximum entropy modeling of short sequence motifs with applications to RNA splicing signals. J. Comput. Biol. *11*, 377–394. https://doi.org/10.1089/1066527041410418.
48. Reese, M.G., Eeckman, F.H., Kulp, D., and Haussler, D. (1997). Improved splice site detection in genie. J. Comput. Biol. *4*, 311–323. https://doi.org/10.1089/cmb.1997.4.311.
49. Schubach, M., Maass, T., Nazaretyan, L., Röner, S., and Kircher, M. (2024). CADD v1.7: Using protein language models, regulatory CNNs and other nucleotide-level scores to improve genome-wide variant predictions. Nucleic Acids Res. *52*, D1143–D1154. https://doi.org/10.1093/nar/gkad989.
50. Ioannidis, N.M., Rothstein, J.H., Pejaver, V., Middha, S., McDonnell, S.K., Baheti, S., Musolf, A., Li, Q., Holzinger, E., Karyadi, D., et al. (2016). REVEL: An ensemble method for predicting the pathogenicity of rare missense variants. Am. J. Hum. Genet. *99*, 877–885. https://doi.org/10.1016/j.ajhg.2016.08.016.
51. Kassahn, K.S., Dang, V.T., Wilkins, S.J., Perkins, A.C., and Ragan, M.A. (2009). Evolution of gene function and regulatory control after whole-genome duplication: Comparative analyses in vertebrates. Genome Res. *19*, 1404–1418. https://doi.org/10.1101/gr.086827.108.
52. Kimmel, C.B., Ballard, W.W., Kimmel, S.R., Ullmann, B., and Schilling, T.F. (1995). Stages of embryonic development of the zebrafish. Dev. Dyn. *203*, 253–310. https://doi.org/10.1002/aja.1002030302.
53. Hill, A.J., Bello, S.M., Prasch, A.L., Peterson, R.E., and Heideman, W. (2004). Water permeability and TCDD-induced edem5a in zebrafish early-life stages. Toxicol. Sci. *78*, 78–87. https://doi.org/10.1093/toxsci/kfh056.
54. Novorol, C., Burkhardt, J., Wood, K.J., Iqbal, A., Roque, C., Coutts, N., Almeida, A.D., He, J., Wilkinson, C.J., and Harris, W.A. (2013). Microcephaly models in the developing zebrafish retinal neuroepithelium point to an underlying defect in metaphase progression. Open Biol. *3*, 130065. https://doi.org/10.1098/rsob.130065.
55. Audain, E., Wilsdon, A., Dombrowsky, G., Sifrim, A., Breckpot, J., Perez-Riverol, Y., Loughna, S., Daly, A., Antoniou, P., Hofmann, P., et al. (2023). Assessing the contribution of rare variants to congenital heart disease through a large-scale

case-control exome study. Preprint at medRxiv. https://doi.org/10.1101/2023.12.23.23300495.

56. Hüffmeier, U., Kraus, C., Reuter, M.S., Uebe, S., Abbott, M.A., Ahmed, S.A., Rawson, K.L., Barr, E., Li, H., Bruel, A.L., et al. (2021). EIF3F-related neurodevelopmental disorder: Refining the phenotypic and expanding the molecular spectrum. Orphanet J. Rare Dis. *16*, 136. https://doi.org/10.1186/s13023-021-01744-1.
57. Martin, H.C., Jones, W.D., McIntyre, R., Sanchez-Andrade, G., Sanderson, M., Stephenson, J.D., Jones, C.P., Handsaker, J., Gallone, G., Bruntraeger, M., et al. (2018). Quantifying the contribution of recessive coding variation to developmental disorders. Science *362*, 1161–1164. https://doi.org/10.1126/science.aar6731.
58. Xia, Z., Tong, X., Liang, F., Zhang, Y., Kuok, C., Zhang, Y., Liu, X., Zhu, Z., Lin, S., and Zhang, B. (2013). Eif3ba regulates cranial neural crest development by modulating p53 in zebrafish. Dev. Biol. *381*, 83–96. https://doi.org/10.1016/j.ydbio.2013.06.009.
59. Sauka-Spengler, T., and Bronner, M. (2010). Snapshot: Neural crest. Cell *143*, 486–486.e1. https://doi.org/10.1016/j.cell.2010.10.025.
60. Jerome, L.A., and Papaioannou, V.E. (2001). DiGeorge syndrome phenotype in mice mutant for the T-box gene, Tbx1. Nat. Genet. *27*, 286–291. https://doi.org/10.1038/85845.
61. Piotrowski, T., Ahn, D.g., Schilling, T.F., Nair, S., Ruvinsky, I., Geisler, R., Rauch, G.J., Haffter, P., Zon, L.I., Zhou, Y., et al. (2003). The zebrafish van gogh mutation disrupts tbx1, which is involved in the DiGeorge deletion syndrome in humans. Development *130*, 5043–5052. https://doi.org/10.1242/dev.00704.
62. Stennard, F.A., Costa, M.W., Lai, D., Biben, C., Furtado, M.B., Solloway, M.J., McCulley, D.J., Leimena, C., Preis, J.I., Dunwoodie, S.L., et al. (2005). Murine T-box transcription factor Tbx20 acts as a repressor during heart development, and is essential for adult heart integrity, function and adaptation. Development *132*, 2451–2462. https://doi.org/10.1242/dev.01799.
63. Lu, F., Langenbacher, A., and Chen, J.N. (2017). Tbx20 drives cardiac progenitor formation and cardiomyocyte proliferation in zebrafish. Dev. Biol. *421*, 139–148. https://doi.org/10.1016/j.ydbio.2016.12.009.
64. Holtzinger, A., and Evans, T. (2005). Gata4 regulates the formation of multiple organs. Development *132*, 4005–4014. https://doi.org/10.1242/dev.01978.
65. Molkentin, J.D., Lin, Q., Duncan, S.A., and Olson, E.N. (1997). Requirement of the transcription factor GATA4 for heart tube formation and ventral morphogenesis. Genes Dev. *11*, 1061–1072. https://doi.org/10.1101/gad.11.8.1061.
66. Dorsett, D., and Krantz, I.D. (2009). On the molecular etiology of Cornelia de Lange syndrome. Ann. N. Y. Acad. Sci. *1151*, 22–37. https://doi.org/10.1111/j.1749-6632.2008.03450.x.
67. Jongmans, M.C.J., Admiraal, R.J., van der Donk, K.P., Vissers, L.E.L.M., Baas, A.F., Kapusta, L., van Hagen, J.M., Donnai, D., de Ravel, T.J., Veltman, J.A., et al. (2006). CHARGE syndrome: The phenotypic spectrum of mutations in the CHD7 gene. J. Med. Genet. *43*, 306–314. https://doi.org/10.1136/jmg.2005.036061.
68. Tartaglia, M., Kalidas, K., Shaw, A., Song, X., Musat, D.L., van der Burgt, I., Brunner, H.G., Bertola, D.R., Crosby, A., Ion, A., et al. (2002). PTPN11 mutations in Noonan syndrome: molecular spectrum, genotype-phenotype correlation, and phenotypic heterogeneity. Am. J. Hum. Genet. *70*, 1555–1563. https://doi.org/10.1086/340847.
69. Fujii, K., Zhulyn, O., Byeon, G.W., Genuth, N.R., Kerr, C.H., Walsh, E.M., and Barna, M. (2021). Controlling tissue patterning by translational regulation of signaling transcripts through the core translation factor eIF3c. Dev. Cell *56*, 2928–2937.e9. https://doi.org/10.1016/j.devcel.2021.10.009.
70. Mestre-Fos, S., Ferguson, L., Trinidad, M.I., Ingolia, N.T., and Cate, J.H.D. (2025). eIF3 engages with 3'-UTR termini of highly translated mRNAs. eLife *13*, RP102977. https://doi.org/10.7554/eLife.102977.

The American Journal of Human Genetics, Volume *112*

Supplemental information

A cardiovascular, craniofacial, and neurodevelopmental disorder caused by loss-of-function variants in the eIF3 complex component genes *EIF3A* and *EIF3B*

Esra Erkut, Cherith Somerville, Marci L.B. Schwartz, Laura McDonald, Qiliang Ding, Olivia M. Moran, Xin Chen, Roozbeh Manshaei, Anne-Sophie Riedijk, Marie-Therese Schnürer, Daniel C. Koboldt, Stylianos E. Antonarakis, Emma C. Bedoukian, Xavier Blanc, Laura K. Conlin, Helen Cox, Karin E.M. Diderich, Bri Dingmann, Christèle Dubourg, Frances Elmslie, Luis F. Escobar, Rachel Gosselin, Maria J. Guillen Sacoto, Cynthia D. Haag, Lisa Herzig, Ramanand Jeeneea, Priti Kenia, Konstantinos Kolokotronis, Anna M. Kopps, Christin Kupper, Hayley Lees, Jacqueline Leonard, Jonathan Levy, Rebecca Littlejohn, Demian Mayer, Scott D. McLean, Nikhil Pattani, Laurence Perrin, Véronique Pingault, Chloé Quelin, Emmanuelle Ranza, Anita Rauch, Sara L. Reichert, Joana Rosmaninho-Salgado, Cara Skraban, Sérgio Sousa, Melissa Stuebben, Paolo Zanoni, Raymond H. Kim, Ian C. Scott, and Rebekah K. Jobling

Supplemental Information

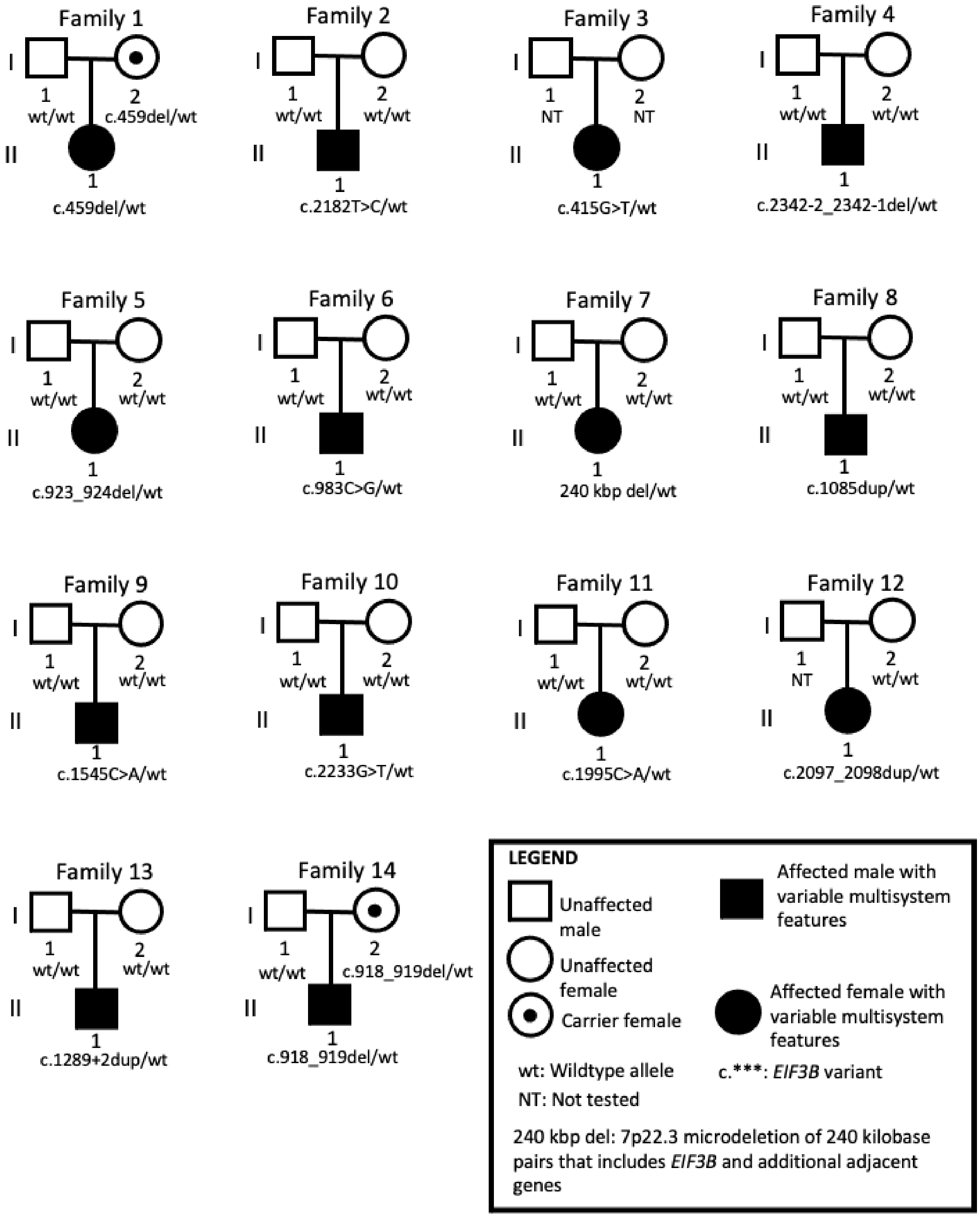

Figure S1. Family pedigrees for individuals with *EIF3B* variants. Solid black symbols represent affected family members with variable multisystem features, primarily including craniofacial differences, heart anomalies, and mild neurodevelopmental symptoms. A dot in the center indicates variant carriers who do not present with clinical symptoms. Family numbers correspond to the proband numbers in the main text and in Tables 2-3.

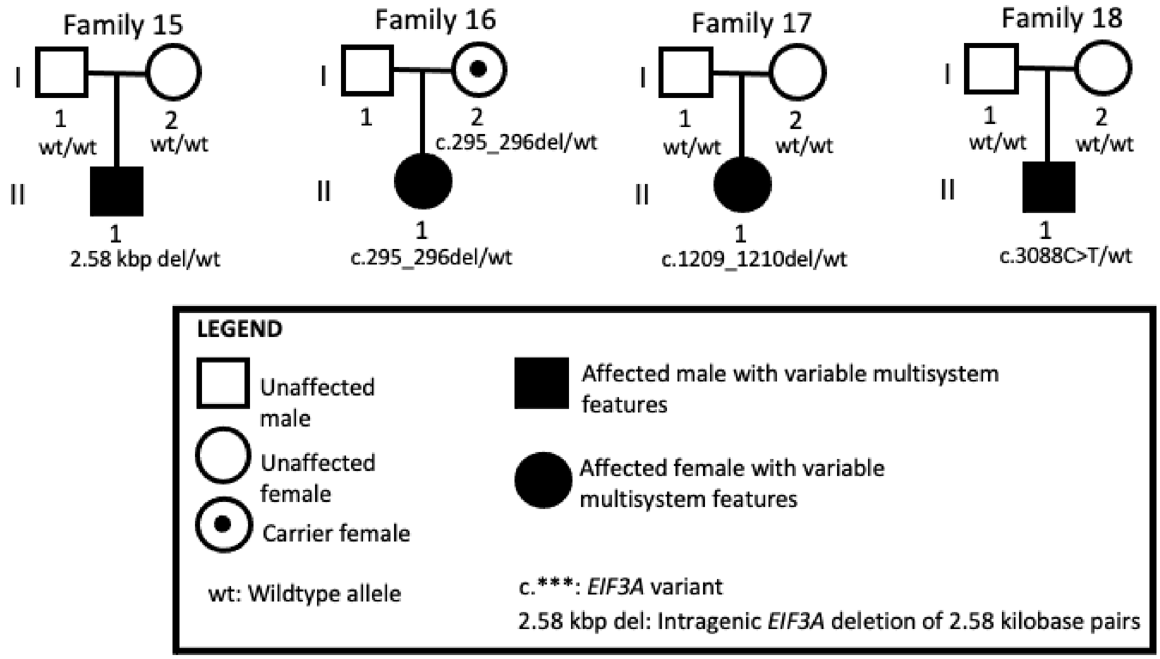

Figure S2. Family pedigrees for individuals with *EIF3A* variants. Solid black symbols represent affected family members with variable multisystem features, primarily including craniofacial differences, heart anomalies, and mild neurodevelopmental symptoms. A dot in the center indicates variant carriers who do not present with clinical symptoms. Family numbers correspond to the proband numbers in the main text and in Tables 2-3.

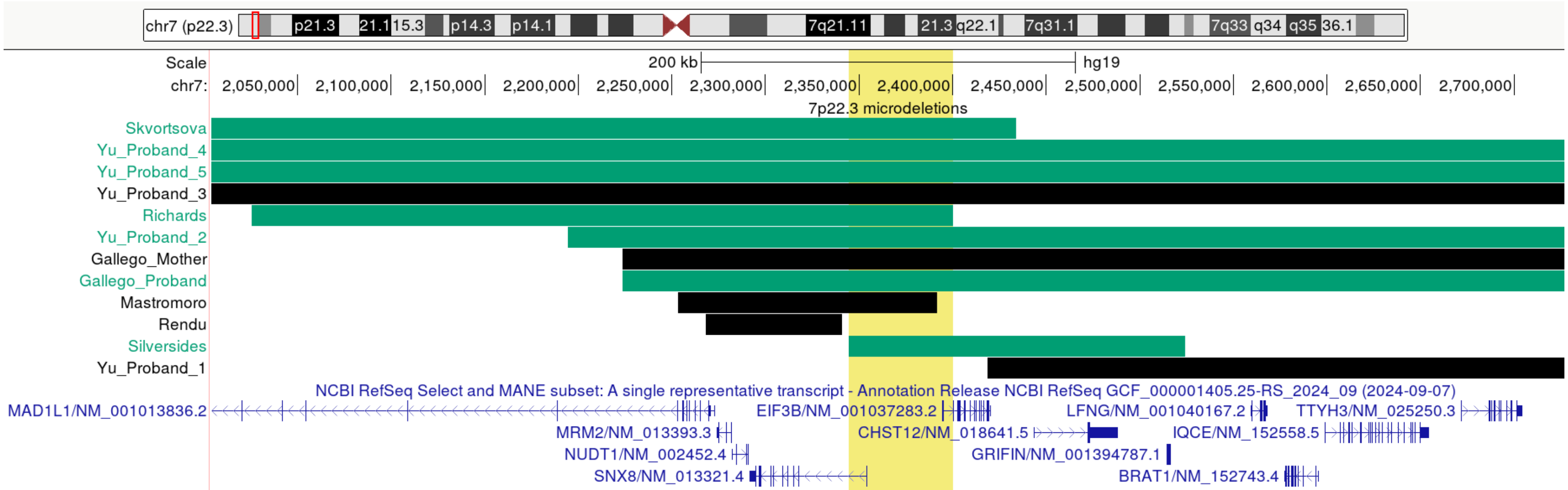

Figure S3. Overlap of 7p22.3 microdeletions reported in the literature. Previously reported deletions overlapping the 7p22.3 region[1-7] are summarized in Table S2. Deletions observed in individuals with cardiac abnormalities (n=7) are marked in green, while those in individuals without cardiac abnormalities are in black. Yellow vertical lines indicate the smallest overlapping region associated with the cardiac phenotype, which includes *EIF3B.* Mapping and gene content were generated using UCSC Genome Custom Tracks Tool[8].

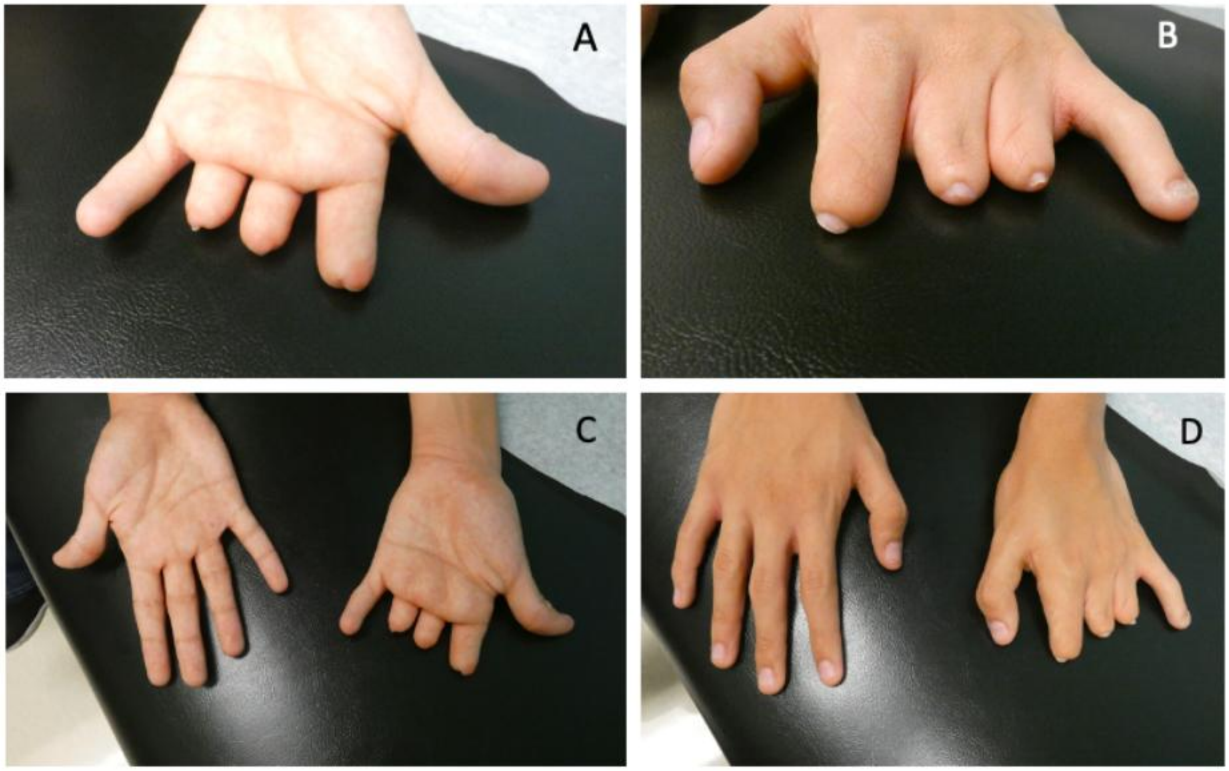

Figure S4 (A-D) Images of the hands of proband #9 presenting with transverse terminal deficiency of the left hand affecting digits 2 through 4 at the distal aspects of the proximal phalanges, with preservation of rudimentary nails.

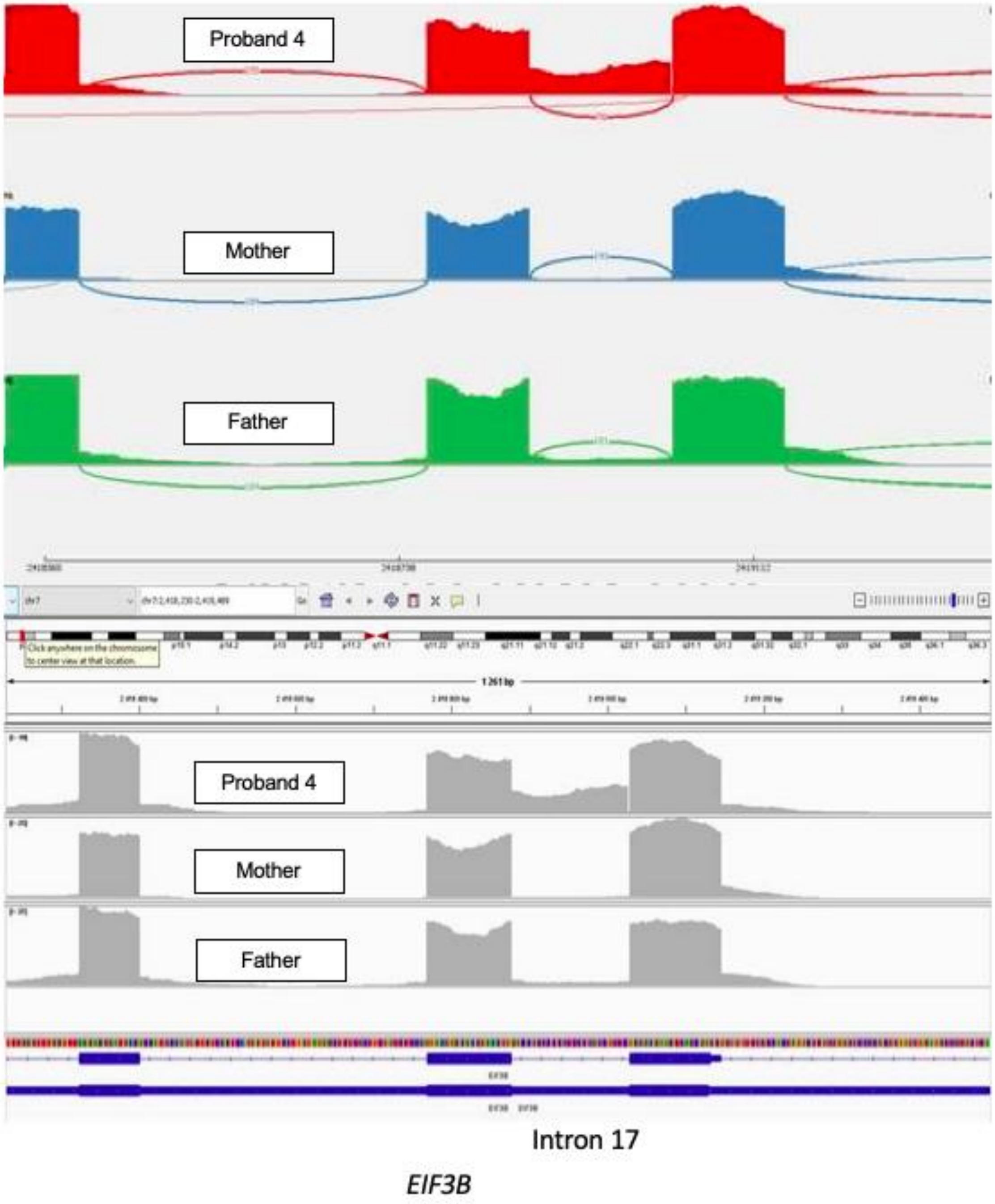

Figure S5: Sashimi plot and coverage graph from IGV for RNA-sequencing results of c.2342-2_2342-1del in proband #4, as well as their mother and father. RNA-seq was performed following identification of a de novo variant via next-generation sequencing (c.2342-2_2342-1del). The sequencing results show intron 17 retention in the proband 4, leading to a readthrough intron starting at amino acid 782 and causing a frameshift. The 2bp deletion is also visible, confirming that intron retention comes from the mutated allele only.

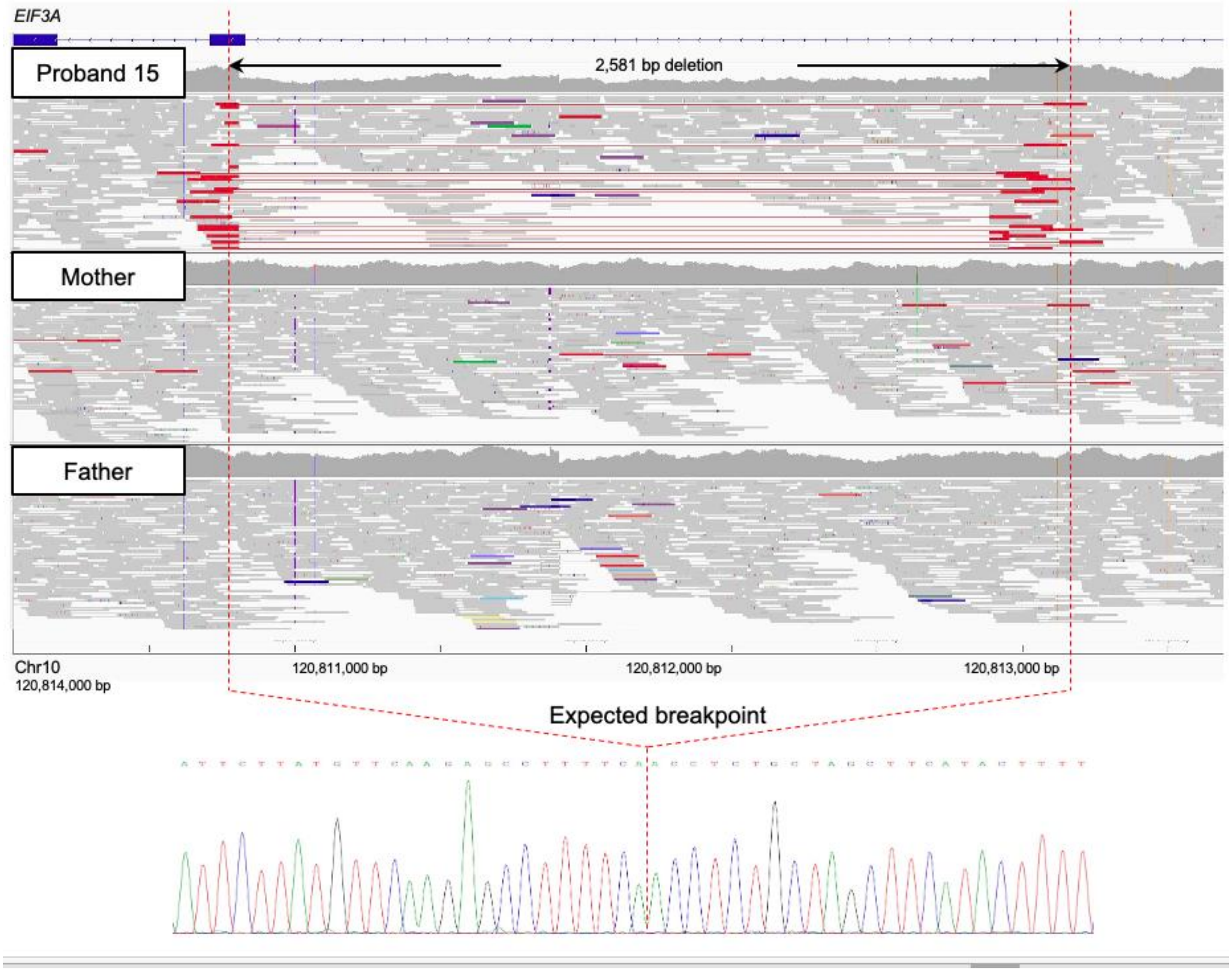

Figure S6: Sanger sequencing validation of the 2.58 kilobase pairs intragenic *EIF3A* deletion in proband #15. IGV browser view of the BAM file for proband #15 and the proband's mother and father, showing the Sanger track confirming the breakpoint. The genomic coordinates for this intragenic deletion are chr10:119051299-119053879 (GRCh38); (chr10:120810811-120813391 [GRCh37]). Sanger validation confirmed that the variant is de novo, with the 5' breakpoint located in intron 14 and the 3' breakpoint extending 23 base pairs into the 5'end of exon 15, disrupting the canonical acceptor splice site of exon 15.

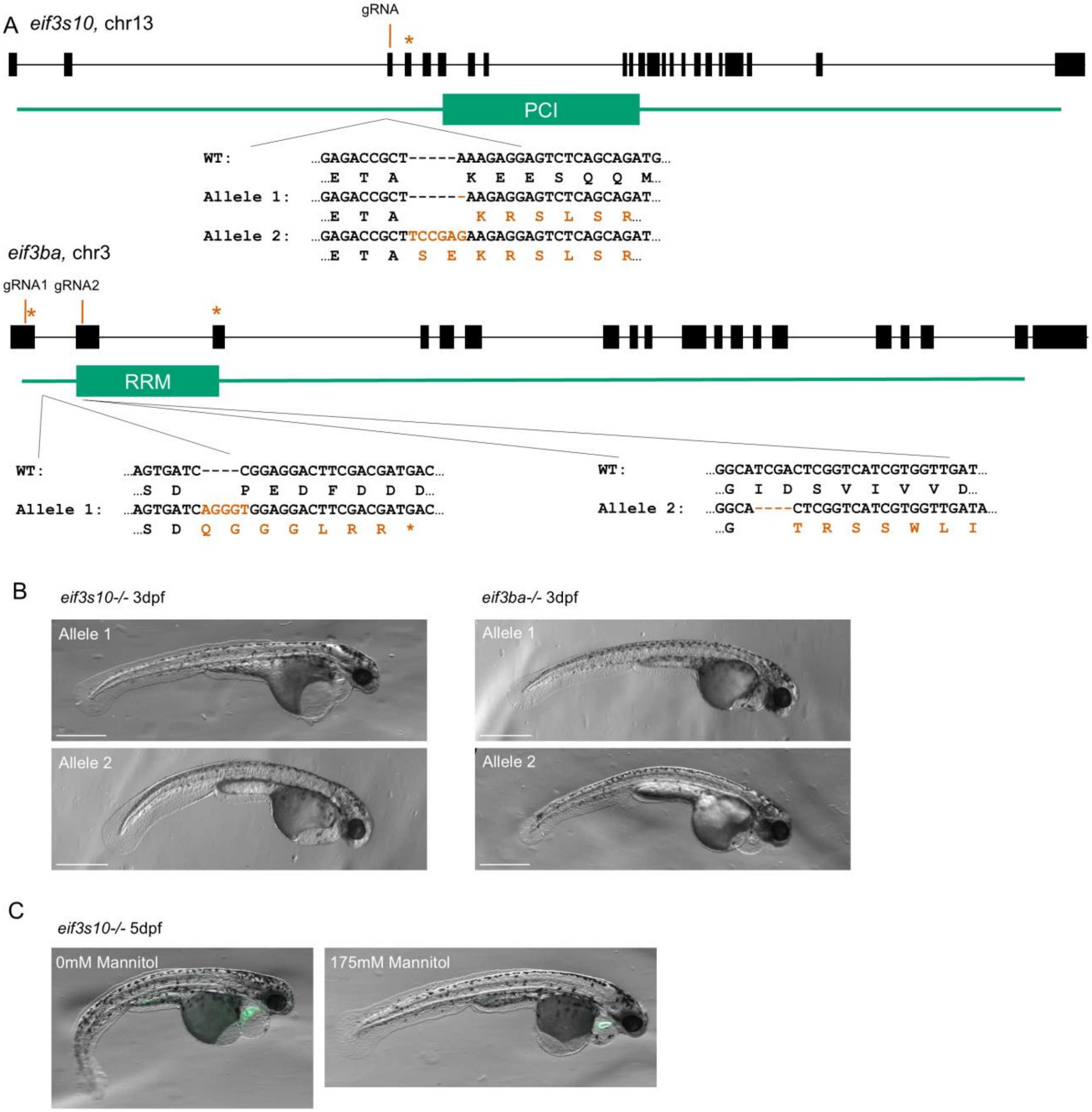

Figure S7: Zebrafish mutant models for *eif3s10* and *eif3ba* exhibit cardiac defects (A) schematic of *eif3s10* and *eif3ba* null mutant generation using the CRISPR/Cas9 system. Black boxes indicate exons, drawn to scale. Orange letters indicate indels generated, and asterisks represent a premature stop codon, which occurs before or inside the critical protein domains (schematized in green). (B) Two different CRISPR alleles for *eif3s10* and *eif3ba* demonstrate the same phenotype. (C) Raising homozygous mutant embryos in 175mM mannitol salts to balance osmolarity did not alleviate the stretched heart phenotype, indicating a primary defect in heart development.

Proband	Gene	GenBank Transcripts	Variant	Zygosity; inheritance	Disease Association	Inheritance Pattern	Classification; criteria applied[a]	Additional evidence
2	*KCNK4* (MIM: 605720)	NM_033310.3; NP_201567.1	c.724G>A, p.(Gly242Ser)	Heterozygous	Facial dysmorphism, hypertrichosis, epilepsy, intellectual disability/developmental delay, and gingival overgrowth syndrome (MIM: 618381)	AD	VUS: PM6, PP3	Identified as *de novo* in this individual; Present in 7 alleles in gnomAD V4.1.0; Not reported in the literature.
2	*PLOD1* (MIM: 153454)	NM_000302.4; NP_000293.2	c.244A>T, p.(Lys82*)	Heterozygous	Kyphoscoliotic type 1 Ehlers-Danlos syndrome (MIM: 225400)	AR	Likely pathogenic: PVS1, PM2	Present in 1 allele in gnomaD V4.1.0; Not reported in the literature. Biallelic loss-of-function in the *PLOD1* gene is an established mechanism of disease.
5	*GJB2* (MIM: 121011)	NM_004004.6; NP_003995.2	c.35delG, p.(Gly12Valfs*2)	Heterozygous	Deafness (MIM: 601544, 220290)	AR, AD	Pathogenic for AR hearing loss: PVS1, PM3_VS, PS4, BA1	ClinVar has an entry for this variant (VCV000017004); classified as pathogenic by the ClinGen Hearing Loss Variant Curation Expert Panel.
5	*TMPRSS3* (MIM: 605511)	NM_001256317.3; NP_001243246.1	c.413C>A, p.(Ala138Glu)	Heterozygous	Deafness (MIM: 601072)	AR	Pathogenic: PS4, PM3_VS, PP1_Strong	ClinVar has an entry for this variant (VCV000046119).
6	*BRAF* (MIM: 164757)	NM_001374258.1; NP_001361187.1	c.1325C>G, p.(Pro442Arg)	Heterozygous; maternal	Noonan syndrome 7 (MIM: 613706)	AD	VUS: No criteria applied (per BRAF RASopathy	Identified in 15 alleles in gnomAD V4.1.0. Not found in a known critical

							ClinGen guidelines)	domain. ClinVar has an entry for this variant (VCV000560678). Not reported in the literature.
6	*ZMIZ1* (MIM: 607159)	NM_020338.4; NP_065071.1	c.767G>C, p.(Gly256Ala)	Heterozygous; maternal	Neurodevelopmental disorder with dysmorphic facies and distal skeletal anomalies (MIM: 618659)	AD	VUS: No criteria applied.	Identified in 9 alleles tested from gnomAD V4.1.0. ClinVar has an entry for this variant (VCV002640635). Not reported in the literature.
6	*MED13* (MIM: 618009)	NM_005121.3; NP_005112.2	c.3676C>T, p.(Arg1226Cys)	Heterozygous; paternal	Intellectual developmental disorder (MIM: 618009)	AD	VUS: BP1	Identified in 28 alleles tested from gnomAD V4.1.0. ClinVar has an entry for this variant (VCV003293987). Not reported in the literature.
18	*SHOC2* (MIM: 602775)	NM_007373.3; NP_031399.2	c.126A>T, p.(Glu42Asp)	Heterozygous; maternal. Also inherited in twin brother.	Noonan syndrome-like with loose anagen hair 1 (MIM: 607721)	AD	VUS: PM2_Supporting, BP4	Absent in gnomAD v4.1.0. ClinVar has an entry for this variant (VCV002200060). Not reported in the literature

Table S3: Additional genetic findings in individuals in the cohort. [a] Classification and criteria based on Richards et al.[9]. Abbreviations: AD: autosomal dominant. AR: Autosomal recessive.

Gene	pLI	LEOUF
EIF3B (MIM: 603917)	1	0.11
AMZ1 (MIM: 615168)	0	1.88
AP5Z1 (MIM: 613653)	0	1.64
BRAT1 (MIM: 614506)	0	1.39
CARD11 (MIM: 607210)	1	0.36
CHST12 (MIM: 610129)	0	1.12
FOXK1 (MIM: 616302)	0.05	0.66
GNA12 (MIM: 604394)	0.93	0.56
GRIFIN (MIM: 619187)	0	1.89
IQCE (MIM: 617631)	0	1.44
LFNG (MIM: 602576)	0.2	0.76
*MAD1L1 (*MIM: 602686)	0	1.23
MMD2 (MIM: 624581)	0	1.35
MRM2 (MIM: 606906)	0	1.72
NUDT1 (MIM: 600312)	0	1.74
PAPOLB (MIM: 607436)	0	1.05
RADIL (MIM: 611491)	0	1.49
RBAK (MIM: 608191)	0	0.75
SDK1 (MIM: 607216)	0	0.77
SNX8 (MIM: 614905)	0	0.74
TTYH3 (MIM: 608919)	0.01	0.68

Table S4. Loss-of-function constraint metrics for genes in the 7p22.3 region. The Probability of being loss-of-function intolerant (pLI) and loss-of-function observed/expected upper bound fraction scores (LEOUF) were obtained from gnomAD v4.1.0. Genes in the 7p22.3 region considered constrained against loss-of-function variation include *EIF3B* and *CARD11*.

References (Table S1-S4)
1. Gallego, C.J., Grant, J., Mikhail, F.M., Barger, C., Robin, N.H. (2010). Use of array comparative genome hybridization in orofacial clefting. J Craniofac Surg. *21*(5), 1591–1594. doi: 10.1097/SCS.0b013e3181ebcc9c.
2. Richards, E.G., Zaveri, H.P., Wolf, V.L., Kang, S.L., Scott, D.A. (2011). Delineation of a less than 200 kb minimal deleted region for cardiac malformations on chromosome 7p22. Am J Med Genet A. *155A*(7), 1729–1734. doi: 10.1002/ajmg.a.34041.
3. Silversides, C.K., Lionel, A.C., Costain, G., Merico, D., Migita, O., Liu, B., Yuen, T., Rickaby, J., Thiruvahindrapuram, B., Marshall, C.R. (2012). Rare copy number variations in adults with tetralogy of Fallot implicate novel risk gene pathways. PLoS Genet. *8*(8), e1002843. doi: 10.1371/journal.pgen.1002843.
4. Rendu, J., Satre, V., Testard, H., Devillard, F., Vieville, G., Fauré, J., Amblard, F., Jouk, P-S. Coutton, C. (2014). 7p22.3 microdeletion disrupting SNX8 in a patient presenting with intellectual disability but no tetralogy of Fallot. Am J Med Genet A. *164A*(8), 2133–2135. doi: 10.1002/ajmg.a.36566.
5. Yu, A.C., Zambrano, R.M., Cristian, I., Price, S., Bernhard, B., Zucker, M., Venkateswaran, S., McGowan-Jordan, J., Armour, C.M. (2017). Variable developmental delays and characteristic facial features-A novel 7p22.3p22.2 microdeletion syndrome? Am J Med Genet A. *173*(6), 1593–1600. doi: 10.1002/ajmg.a.38241.
6. Mastromoro, G., Capalbo, A., Guido, C.A., Torres, B., Fabbretti, M., Traversa, A., Giancotti, A., Ventriglia, F., Bernardini, L., Spalice, A. et al. (2020). Small 7p22.3 microdeletion: Case report of Snx8 haploinsufficiency and neurological findings. Eur J Med Genet. *63*(4), 103772. doi: 10.1016/j.ejmg.2019.103772.
7. Skvortsova, L., Perfilyeva, A., Bespalova, K., Kuzovleva, Y., Kabysheva, N., Khamdiyeva, O. (2024). 7p22.3 microdeletion: A case study of a patient with congenital heart defect, neurodevelopmental delay and epilepsy. Orphanet J Rare Dis. *19*(1), 301–308. doi: 10.1186/s13023-024-03321-8.
8. Perez, G., Barber, G.P., Benet-Pages, A., Casper, J., Clawson, H., Diekhans, M., Fischer, C., Navarro Gonzalez, J., Hinrichs, A.S., Lee, C.M., et al. (2025). The UCSC genome browser database: 2025 update. Nucleic Acids Res. 53(D1), D1243–D1249. doi: 10.1093/nar/gkae974.
9. Richards, S., Aziz, N., Bale, S., Bick, D., Das, S., Gastier-Foster, J., Grody, W.W., Hegde, M., Lyon, E., Spector, E., et al. (2015). Standards and guidelines for the interpretation of sequence variants: a joint consensus recommendation of the American College of Medical Genetics and Genomics and the Association for Molecular Pathology. Genet Met 17(5), 405-424. doi: 10.1038/gim.2015.30.
